# Peripheral inflammatory effects of different interventions for treatment-resistant depression: A systematic review

**DOI:** 10.1016/j.nsa.2022.101014

**Published:** 2022-11-01

**Authors:** Rebecca Strawbridge, Estefany Izurieta, Elana Day, Helena Tee, Kate Young, Co Co Tong, Allan H. Young, Anthony J. Cleare

**Affiliations:** a Department of Psychological Medicine, Institute of Psychiatry, Psychology & Neuroscience, King's College London, London, UK; b School of Nursing, King's College London, London, UK

**Keywords:** cytokine, Inflammation, Electroconvulsive therapy, Ketamine, Bupropion, Lithium, Aripiprazole, Quetiapine, Systematic review, Biomarker

## Abstract

**Background:**

Immune-related mechanisms are increasingly recognised as important in the pathophysiology of depression, particularly treatment-resistant depression (TRD). Preliminary evidence suggests that people with TRD may benefit from anti-inflammatory interventions; however, the relative anti-inflammatory effects of therapies currently recommended for TRD have not been integrated in a specific evidence synthesis.

**Methods:**

A systematic search was performed to identify articles published up to February 8, 2021. Results from databases (Pubmed, Embase) and handsearches were reviewed to include any longitudinal study examining circulating marker(s) of inflammation in humans before and after treatment with electroconvulsive therapy (ECT), ketamine, bupropion, lithium, aripiprazole, or quetiapine. We undertook a narrative synthesis of results and risk of bias assessment to incorporate and interpret the evidence.

**Results:**

Of 57 included studies, 34 assessed the effects of ketamine and 12 of ECT, with other medications examined in ≤5 studies each. Results were highly heterogeneous. Numerically, more frequent increases than decreases in pro-inflammatory markers were reported after ketamine, quetiapine and lithium (although more frequent *significant* decreases were observed for lithium). Findings for ECT and bupropion were even more inconsistent. Two studies consistently reported inflammatory reductions (significant/non-significant) after aripiprazole.

**Conclusions:**

Treatment effects on inflammatory proteins are confounded by several factors: ketamine, in most studies, was co-administered with other surgical procedures, while ketamine and ECT findings are also obscured by variable post-procedure assessment timing. The evidence base for other treatments is scant. Greater examination is warranted to establish whether inflammatory biomarkers could be used to stratify TRD patients to a ‘most appropriate’ intervention.

## 1 Introduction

Enhancing treatment for major depressive disorder (MDD) is urgently required: in addition to its high prevalence and disabling impacts, a high proportion of people affected by MDD do not respond adequately to first-line treatments and therefore suffer enduring symptoms (Jaffe et al., 2019). Furthermore, they are less likely to respond to subsequent interventions (Taylor et al., 2021a) and up to one third of patients experience substantive treatment resistant depression (TRD) (Nemeroff, 2007). Providing interventions that are more likely to be successful, at the earliest possible stage of illness, is fundamental in alleviating the burden of MDD.

Considerable research efforts are now focusing on a pathway towards precision medicine, attempting to establish patient characteristics which predict a good clinical response to specific intervention options (Taylor et al., 2019; Pisoni et al., 2020). To date, research attempting advancement towards truly individualised medicine has been challenged by inconsistent findings and insufficient replication. It has been suggested that a stratified treatment model may be a more feasible first step towards implementable precision psychiatry (Arns et al., 2022). Here, participants would be sub-grouped according to factors that are differentially affected by the treatments to be selected from.

A promising focus for treatment stratification in TRD is using biomarkers of immune-inflammatory dysregulation. Its role in the pathophysiology of depression broadly is increasingly established, and we outline below several strands of evidence which reinforce inflammation as a potentially valuable domain for identifying ways to treat people with TRD more effectively.

1. Aberrant inflammatory biomarkers are present in a subset of, but not all, people experiencing depression (Krishnadas and Cavanagh, 2012).
2. Elevated pro-inflammatory biomarkers frequently predict subsequent non-response to first-line treatments for MDD, notably monoaminergic antidepressants (Liu et al., 2019; Strawbridge et al., 2015) and psychological therapies (Strawbridge et al., 2020).
3. Accordingly, it has been suggested that people with TRD could represent an ‘inflammatory’ subgroup of MDD (Raison et al., 2013; Strawbridge et al., 2018a).
4. Elevated pro-inflammatory markers are also associated with various measures of depressive illness severity, including retrospectively, cross-sectionally and prospectively in samples of people with TRD (Strawbridge et al., 2019a).
5. Specific anti-inflammatory medications have been heralded as possible treatment options to improve outcomes for people with mood disorders, particularly for individuals with elevated pro-inflammatory biomarkers (Jones et al., 2020; Nettis et al., 2021).
6. Although antidepressants can reduce inflammation (Gałecki et al., 2018), there is evidence that this does not manifest in people not responding and first-line antidepressants do not have pronounced direct effects on inflammatory pathways (Strawbridge et al., 2015).
7. Treatments recommended for people with TRD have more diverse mechanisms than first-line antidepressants, some of which are purported to be anti-inflammatory (Yang et al., 2019). This is expanded upon below.

TRD treatments recommended across guidelines include second-generation antipsychotics [SGAs] (i.e., aripiprazole, quetiapine), the unique mood stabilizer lithium, the anaesthetic ketamine, neuro-stimulatory electroconvulsive therapy (ECT) and the atypical antidepressant bupropion (Taylor et al., 2020, 2021b; Fekadu et al., 2018). Bupropion is not universally considered 1st line, but is listed as such in the most recent guidelines (Taylor et al., 2020) alongside strong meta-analytic evidence lacking for other recommended TRD interventions including the SGAs olanzapine and risperidone, anticonvulsant mood stabilisers, thyroid hormones and stimulants, which tend not to be recommended as first-line augmenters due to small evidence bases, small effect sizes or acceptability drawbacks (Taylor et al., 2020; Scott et al. 2022). Although ketamine and ECT are rarely considered 1st line (for reasons including cost of administration and monitoring, risks and longevity of response) we consider these key TRD therapies in this review, due to high evidence strength (Scott et al. 2022), efficacy (Carter et al., 2020; Kirov et al., 2021; Strawbridge et al., 2019b) and purported immune pathway mechanisms (Gagne et al., 2022). There are not yet established factors predicting which of these agents are most likely to be successful for particular patients (Taylor et al., 2019).

It has already been proposed that individuals with an inflammatory component to their depressive illness may respond best to interventions with more pronounced anti-inflammatory effects, and those without aberrant inflammation may benefit from other therapies (Strawbridge et al., 2018a; Yang et al., 2019). This was indicated in a recent systematic review which found that people with elevated pro-inflammatory cytokines responded better to treatments that the authors had highlighted as anti-inflammatory, and that inflammation predicted a poorer response to ‘non-anti-inflammatory’ treatments. However, the classification of treatments into ‘anti-inflammatory’ and ‘non-anti-inflammatory’ was not based on hard evidence of the treatments' effects on inflammatory biomarkers (Yang et al., 2019). This is likely because there is a distinct paucity of studies examining the effects on inflammation of these treatments specifically in populations with TRD. Fortunately, there is also data from across disciplines that examines inflammatory effects of these treatments in other conditions and populations, and which could be synthesised alongside data in TRD. No such evidence synthesis to our knowledge has yet been undertaken.

### 1.1 Aims and objectives

This systematic review aimed to synthesise the current evidence investigating the impact of TRD recommended interventions on inflammatory markers in humans. Specifically, we aimed to identify all longitudinal studies reporting peripheral inflammatory marker levels before and after (at least) one of our pre-selected interventions in any human population. The treatments examined were ECT, ketamine, lithium, quetiapine, aripiprazole and bupropion.

## 2 Methods

This review has been conducted after pre-registration of the review protocol on International Prospective Register of Systematic reviews (PROSPERO; ID CRD42021231890) and is reported according to the Preferred Reporting Items for Systematic Reviews and Meta-Analyses (PRISMA) guidelines (Sterne et al., 2016).

### 2.1 Study eligibility criteria

To be included in the review, articles had to report data from original longitudinal studies of humans including information from at least one peripheral inflammatory biomarker (Farrah et al., 2019) before the intervention and comparable data after the intervention. To maximise inclusivity, any age and diagnosis of participants was permitted, as was any intensity (dose; duration) of treatment. Comparator interventions were not specifically focused on in the review although studies were not excluded on the basis of randomisation/controlled trial design or comparator treatments. We included articles from any year and in any language. For articles not written in English, data were extracted by one reviewer (EI) using internet translation software and checked by a native speaker (CCT).

### 2.2 Systematic search procedure

Relevant articles were identified by systematic search of the electronic databases PubMed and EMBASE (from inception to February 8, 2021). The search terms used were: (cytokine∗ OR inflammation OR inflammatory) AND (electroconvulsive therapy OR ECT OR ketamine OR esketamine OR bupropion OR lithium OR aripiprazole OR quetiapine) [Title/Abstract]. All articles identified from searches were subject to title and abstract screening by two reviewers (EI and HT or ED). Any article not definitively excluded at this stage was subject to a review of the full text by two authors independently. Handsearching was also undertaken (by EI) searching reference lists of relevant review articles. The two independent reviewers were blind to one another's decision ratings. Subsequently, any discrepancies between reviewers were highlighted (RS) and consensus was reached through discussion (with RS, AJC).

During this process, one amendment to the protocol was made: during the title/abstract screening phase, it was decided upon consensus that animal, in vitro and ex vivo studies were to be excluded. This was primarily due to the reduced applicability of findings between cells/animals and in vivo in humans. Additionally, because of the large quantity of evidence retrieved, their inclusion would have prevented a high-quality review (i.e., sufficiently synthesising and comparing all data in a timely manner) from being undertaken.

### 2.3 Data extraction procedure

Data extraction of relevant information from included studies was undertaken by EI and checked by KY or CCT. Disagreements in data extraction were resolved as above (discussion and consensus with RS). Extraction was standardised between studies using a pre-developed spreadsheet of relevant variables (including study information, details of design and methodology, participant and intervention characteristics, and primary outcome findings, as well as risk of bias assessment ratings). The primary outcome was the difference between pre- and post-treatment levels of inflammatory markers. In some cases, biomarker measurements were taken at multiple post-treatment timepoints. We selected one using a pre-defined priority order. This was decided upon to minimise any acute post-treatment effects (particularly relevant for ECT and ketamine) but to be not so long after intervention as to increase the influence of non-treatment factors on inflammation. Thus, if available, biomarkers were assessed 24–48 ​h after a last treatment (prioritising later measures within window); if not available, data were taken from the following time-windows after last treatment, in order of preference: 49–120 ​h (prioritising earlier measures within window), <24 ​h (prioritising later measures within window), >120 ​h (prioritising earlier assessments).

### 2.4 Risk of bias (RoB) assessment

RoB for each study was assessed by two authors (EI and CCT or KY), with discrepancies resolved through discussion and consensus with RS. The ROBINS-I tool was used to rate RoB (Sterne et al., 2016) given its applicability to various longitudinal designs; however, this was slightly modified (prior to study data extraction), to account for the nature of this review's questions and different study designs included (e.g., randomisation where applicable). Modifications of this type are considered acceptable where indicated (Farrah et al., 2019), in this case to maximise applicability across the heterogeneous methodologies employed between included studies. Each of the nine domains assessed was categorised as either low, moderate or unclear, or high risk of bias and subsequently an overall rating was given.

### 2.5 Data analysis

Due to the clinical and methodological heterogeneity of included studies, a quantitative analysis was not considered appropriate. Had sufficient number of studies been included we would have attempted to investigate this heterogeneity using pre-defined subgroups (which we have instead explored narratively), however quantitative analysis to do so (e.g., subgroup analysis or meta-regression) are not recommended unless there are sufficient available data (Deeks et al., 2022). Instead, a narrative synthesis was performed using a Synthesis Without Meta-analysis (SWiM) approach (Campbell et al., 2020). Text, tables, and figures were employed to present results including sample sizes, treatment information (including duration; dose), inflammatory markers assessed and their levels before and after treatment. Each treatment was considered separately; within these, studies were grouped by populations investigated and then design (randomised or non-randomised interventional, or naturalistic). We particularly highlighted studies of individuals with mood disorders that were not rated to have a high risk of bias.

## 3 Results

### 3.1 Systematic search

**Fig. 1:**
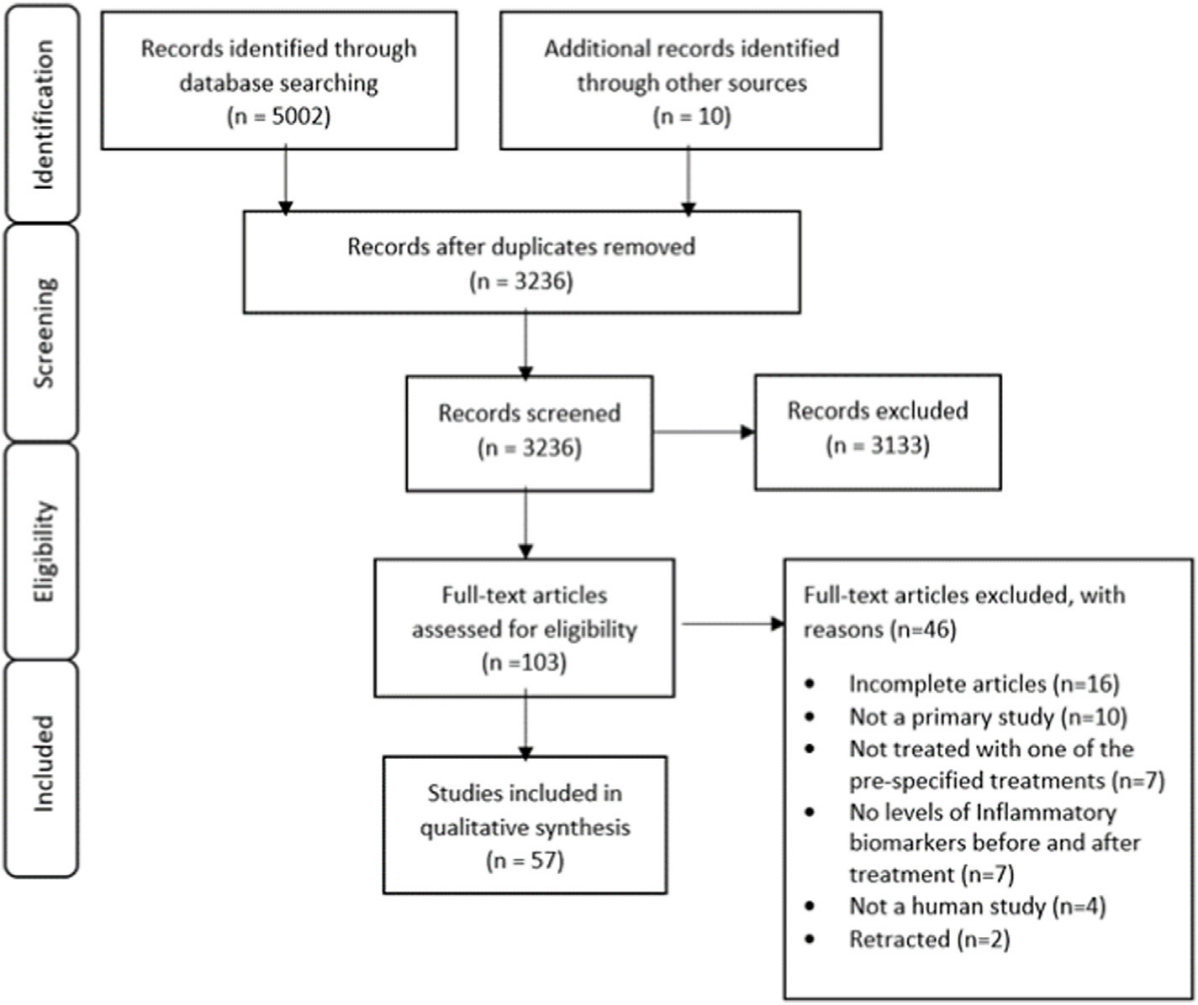
PRISMA flow chart of study inclusion.

5002 articles were initially identified (3236 after removing duplicates). After screening all titles and abstracts, 103 full text articles were reviewed. Of these, 57 were included (Fig. 1).

### 3.2 Characteristics of included studies

A total of 2780 participants were examined. As shown in Table 1, the most frequent interventions studied were ketamine (34 studies, n ​= ​1863 participants) and ECT (12 studies, n ​= ​457 participants). There were 5 studies of quetiapine (n ​= ​177 participants), 4 of lithium (n ​= ​174 participants, including one study combining lithium and quetiapine), 2 of aripiprazole (n ​= ​92 participants) and 2 of bupropion (n ​= ​58 participants).

**Table 1:** Characteristics of included studies.

	Study	Design	Continent	n	% female	Age Range Average		IP/OP	Population
**(es)ketamine studies**	Chen et al. (2018)	RCT	Asia	47	75	nr	48	OP	TRD
	Kiraly et al. (2017)	RCT	NA	59	50	nr	45	IP	TRD
	Mkrtchian et al. (2021)	RCT	NA	58	60	nr	36	IP	TRD
	Park et al.	Nat.	NA	80	51	18–65	44	IP	TRD (unipolar/bipolar)
	Zhan et al. (2020)	NRT	Asia	60	63	18–65	34	IP	MDD
	Zhou et al. (2020)	NRT	Asia	44	nr	18–65	35	IP	MDD
	Wang et al. (2017)	RCT|Q|	Asia	22	41	45–80	58	IP	Acute lung injury
	Akhlagh et al. (2010)	RCT	Asia	50	54	<75	59	IP	Surgery (on-coronary artery bypass)
	Ali and Mokhtar (2017)	RCT	Africa	60	0	>45	67	IP	Surgery (radical prostatectomy)
	Silva et al. (2012)	RCT	SA	29	100	18–60	43	IP	Surgery (abdominal hysterectomy)
	Bhutta et al. (2012)	RCT	NA	24	67	<1	0.42	IP	Surgery (cardiopulmonary bypass)
	Cho et al. (2009)	RCT	Asia	50	32	50–73	61	IP	Surgery (off-coronary artery bypass)
	Cho et al. (2021)	RCT	Asia	100	23	20–80	57	IP	Surgery (colorectal cancer)
	D'Alonzo et al. (2011)	RCT	NA	40	23	<18	12	IP	Surgery (thoracic)
	Du et al. (2011)	RCT	Asia	40	100	18–65	35	IP	Surgery (gynaecological laparoscopic)
	Fiorelli et al. (2015)	RCT	Europe	75	27	>18	59	IP	Surgery (thoracotomy)
	Huang and Liu (2006)	RCT	Asia	30	53	22–58	Nr	IP	Surgery (upper abdominal)
	Hudetz et al. (2009)	RCT	NA	58	0	55–84	68	IP	Surgery (cardiopulmonary bypass)
	Ibrahim et al. (2017)	RCT	Aus	66	12	>5	2	IP	Surgery (for congenital cyanotic heart)
	Kartalov et al. (2012)	RCT	Europe	50	52	nr	58	IP	Surgery (laparoscopic cholecystectomy)
	Kawaguchi et al., 2020	RCT	Asia	60	0	>17	67	IP	Surgery (radical prostatectomy)
	Luggya et al. (2017)	RCT	Africa	39	44	18–70	Nr	IP	Surgery (abdominal/perineal)
	Ma et al. (2013)	RCT	Asia	120	53	>60	66	IP	Surgery (orthopaedic)
	Mostafa et al. (2008)	RCT	Africa	40	nr	18–65	32	IP	Surgery (abdominal)
	Roytblat et al. (1998)	RCT	Asia	31	23	nr	68	IP	Surgery (cardiopulmonary bypass)
	Senapathi et al. (2016)	RCT	Asia	36	100	nr	28	IP	Surgery (emergency caesarean)
	Senapathi et al. (2019)	RCT	Asia	24	0	45–79	62	IP	Surgery (transurethral prostate resection)
	Singh et al. (2020)	RCT	Asia	60	27	nr	62	IP	Surgery (off-coronary artery bypass)
	Tu et al. (2007)	RCT	Europe	37	68	nr	69	IP	Surgery (routine cataract)
	Welters et al. (2011)	RCT	Europe	128	21	62–71	66	IP	Surgery (cardiopulmonary bypass)
	Xie et al. (2015)	RCT	Asia	65	100	55–65	60	IP	Surgery (for cervical cancer)
	Xin et al. (2006)	RCT	Asia	40	40	50–72	40	IP	Surgery (gastric cancer)
	Yang et al. (2006)	RCT	Asia	20	15	nr	Nr	IP	Surgery (orthotopic liver transplant)
	Altiparmak et al. (2018)	NRT	Asia	121	1	nr	48	OP	Surgery (inguinal hernia repair)
**ECT studies**	Zincir et al. (2016)	NRT	Asia	80	65	nr	33	IP	TRD
	Fluitman et al. (2011)	Nat.	Europe	12	75	21–71	Nr	OP	TRD
	Hestad et al. (2003)	RCT	Europe	23	35	33–82	57	IP	MDD
	Järventausta et al. (2017)	NRT	Europe	30	40	28–85	57	IP	MDD
	Kruse et al. (2018)	NRT	NA	40	52	nr	42	IP	MDD
	Kruse et al. (2020)	NRT	NA	40	55	nr	41	IP	MDD
	Lehtimäki et al. (2008)	NRT	Europe	9	67	28–73	55	IP	MDD
	Rush et al. (2016)	Nat.	Europe	81	56	nr	51	IP	Melancholic depression
	Mindt et al. (2020)	Nat.	Europe	12	58	21–83	59	IP	MDD (unipolar/bipolar)
	Belge et al. (2020)	NRT	Europe	62	77	40–65	58	IP	BD depression
	Kargar et al. (2014)	RCT	Asia	48	64	17–70	34	OP	BD (depressed, mixed, manic)
	Kartalci et al. (2016)	RCT	Asia	20	40	nr	29	IP	TRS
**L/Q**^a^	Gao et al. (2018)	RCT	NA	42	52	>17	39	OP	BD (most depressed)
	Li et al. (2015)	RCT	Asia	41	61	18–65	38	OP	BD mania
**Quet.**	Fiedorowicz et al. (2019)	RCT	NA	27	48	18–65	41	OP	BD (type II) depression
	Igue et al. (2011)	NRT	NA	57	18	nr	30	OP	Schizophrenia spectrum ​+ ​SUD
	Kao et al. (2016)	NRT	Asia	28	50	29–60	43	IP	Schizophrenia
**Li.**	Ricken et al. (2018)	Nat.	Europe	95	60	>18	49	OP	MDD
	Merendino et al. (1994)	RCT	Europe	20	100	56–74	65	OP	Breast cancer
**Ari.**	Juncal-Ruiz et al. (2018)	NRT	Europe	75	48	15–50	nr	IP	Psychosis first episode
	Sobiś et al. (2015)	NRT	Europe	17	41	25–80	51	OP	Schizophrenia
**Bup**	Eller et al. (2009)	RCT	Europe	28	61	nr	31	OP	TRD (early stage)
	Tafseer et al. (2021)	Nat.	Asia	30	33	18–60	38	OP	MDD

a Participants treated with lithium OR quetiapine. ECT ​= ​electroconvulsive therapy, Quet. ​= ​quetiapine, Li. ​= ​lithium, Ari. ​= ​aripiprazole, Bup. ​= ​bupropion, RCT ​= ​randomised controlled trial, NRT ​= ​non-randomised trial, Nat. ​= ​naturalistic/observational study, NA = North America, SA = South America, nr ​= ​not reported, IP ​= ​inpatient, OP ​= ​outpatient, TRD ​= ​treatment-resistant depression, MDD ​= ​major depressive disorder, BD ​= ​bipolar disorder, TRS ​= ​treatment-resistant schizophrenia.

Ketamine studies were more likely to be randomised trials (88%), specifically recruit older adult (41%) and/or populations needing medical surgeries (79%) and be conducted in Asia (56%). ECT studies were more likely to be non-randomised trials (50%), from Europe (58%) and recruit patients with mood disorders (8 unipolar studies, 2 bipolar, 1 both, 1 treatment-resistant schizophrenia). Of the lesser-examined treatments, aripiprazole was used to treat only participants with psychosis, while quetiapine was used either for psychosis (2 studies) or bipolar disorders (3 studies), lithium was primarily treating people with bipolar disorder (2 studies) but was also trialled in one MDD (Ricken et al., 2018) and one breast cancer population (Merendino et al., 1994).

### 3.3 Primary outcome

Treatment characteristics are displayed in Table 2, which also includes a summary note of studies where other factors may have influenced a reduction (concomitant treatment with celecoxib in one study) or rise (surgical procedures undertaken alongside ketamine) in inflammatory activity. The most common inflammatory markers measured were CRP, TNFα, IL-6, IL-10, and IL-8. TNFα was the only marker assessed in >1 study for each treatment.

**Table 2:** Treatment characteristics.

	Study	Concomitant tx	Inflammatory markers	Dose/duration	Comparator arms	PO con-found
**(es)ketamine studies**	Chen et al., 2018	TAU	CRP, TNFα, IL6	Single 0.5 ​mg/kg	SAL	∼
	Kiraly et al., 2017	TAU	TNFα, IL: 6, 1β	Single 0.5 ​mg/kg	SAL	∼
	Mkrtchian et al., 2020	TAU	CRP	Single 0.5 ​mg/kg	SAL	∼
	Park et al., 2017	BD: MS	TNFα, IFNγ, IL: 6, 8, 10	Single 0.5 ​mg/kg	SAL	∼
	Zhan et al., 2020	TAU	TNFα, IFNγ, MIP: 1β, 3α, IL: 1β, 6, 8, 10, 13	6 × infusion 0.5 ​mg/kg during surgery	n/a	∼
	Zhan et al., 2020	TAU	TNFα, IFNγ, MIP: 1β, 3α, IL: IL: 1β, 6, 8, 10, 13	6 × infusion 0.5 ​mg/kg during surgery	n/a	∼
	Wang et al., 2017	SAL	IL1β	Aerosol inhalation 1 x ∼5 days	TAU/PRO	∼
	Akhlagh et al., 2010	Surgery	CRP, IL6	1.25 mcg/kg/min during surgery	SAL	PI
	Ali and Mokhtar 2017	Surgery	TNFα, IL6	0.5 ​mg/kg (single/+repeat 0.2 ​mg/kg during surgery)	SAL	PI
	Silva et al., 2012	Surgery	TNFα, IL: 6, 10	0.25 ​mg 30 ​min after surgical incision	n/a	PI
	Bhutta et al., 2012	Surgery	CRP, MIP1α, MCP1, IL: 1α, 1Ra, 6, 8, 10, 13	Single 2 ​mg/kg	SAL	PI
	Cho et al., 2009	Surgery	CRP, IL6, TNFα	Bolus 0.5 ​mg/kg	SAL	PI
	Cho et al., 2021	Surgery	CRP, IL6, TNFα	Single 0.25 ​mg/kg; 0.05 ​mg/kg/hr during surgery	SAL	PI
	D′Alonzo et al., 2011	Surgery	IL6, CRP	Single 0.5 ​mg/kg	SAL	PI
	Du et al., 2011	Surgery	IL6, TNFα	Single 0.25 ​mg/kg	SAL	PI
	Fiorelli et al., 2015	Surgery	CRP	Single 1 ​mg/kg	SAL	PI
	Huang et al., 2006	Surgery	IL6, TNFα, IL8	Single IV 0.2 or 0.6 ​mg/kg	SAL	PI
	Hudetz et al., 2009	Surgery	CRP	Bolus 0.5 ​mg/kg	SAL	PI
	Ibrahim et al., 2017	Surgery	CRP, TNFα, IL: 6, 8, 10	Single 2 ​mg/kg	SAL	PI
	Kartalov et al., 2012	Surgery	TNFα, IL: 6, 1β	Single 0.25 ​mg/kg	SAL	PI
	Kawaguchi et al., 2020	Surgery	TNFα, IL: 1β, 6, 10	1 ​mg/kg during surgery	PRO	PI
	Luggya et al., 2017	Surgery	IL: 1β, 6	Single 0.5 ​mg/kg	SAL	PI
	Ma et al., 2013	(+DM)	IL6	0.5 ​mg/kg during surgery	SAL	PI
	Mostafa et al., 2008	Surgery	IL: 6, 8	0.5 ​mg/kg (single/+ repeat 0.2 ​mg/kg) during surgery	n/a	PI
	Roytblat et al., 1998	Surgery	IL6	Single 0.25 ​mg/kg	SAL	PI
	Senapathi et al., 2016	Surgery	CRP	IV 0.3 ​mg/kg	SAL	PI
	Senapathi et al., 2019	Surgery	CRP, IL: 8, 10	Single 0.15 ​mg/kg	SAL	PI
	Singh et al., 2020	Surgery/alprazolam	CRP, IL6, TNFα	Bolus 0.5 ​mg/kg	SAL	PI
	Tu et al., 2007	Surgery	TNFα, IFNγ, MCP1, IL: 1α, 1β, 6, 8, 10	Infusion 1 ​mg/kg	PRO/ignocaine ​+ ​levobupivacaine	PI
	Welters et al., 2011	Surgery/med	TNFα, IL: 6, 8, 10	Bolus 1–3 ​mg/kg	Sufentanil	PI
	Xie et al., 2015+	Surgery	TNFα, IL: 6, 8	IV 0.3 ​mg/kg, repeat 4 ​μg/kg during surgery	SAL	PI
	Xin et al., 2007+	Surgery	TNFα, IL: 6, 8, 10	IV 1 ​mg/kg -1 ​h during surgery	SAL/PRO	PI
	Yang et al., 2006+	Surgery	TNFα, IL: 6, 10	IV 0.25 ​mg/kg, infusion 0.5 ​mg/kg/h during surgery	SAL	PI
	Altiparmak et al., 2018	Surgery	CRP	IV 0.2–0.3 ​mg/kg during surgery	DM	PI
**ECT studies**	Zincir et al., 2016	n/a	TNFα, IFNγ, IL: 6, 10	Bilateral–bitemporal; 3 ​× ​1.5–4 weeks	SAL	∼
	Fluitman et al., 2011	NR	TNFα, IFNγ, IL: 6, 10	Right unilateral +/bilateral. 2 ​× ​7 weeks	n/a	∼
	Hestad et al., 2003	TAU	TNFα	Right unilateral 3 ​× ​1.5–6 weeks	n/a	∼
	Järventausta et al., 2017	TAU	IL: 1β, 1Ra, 6	Bilateral; 3 × week (10 days)	n/a	∼
	Kruse et al., 2018	n/a	TNFα, CRP, IL: 6, 8	Right unilateral/bilateral; 3 ​× ​1 week	n/a	∼
	Kruse et al., 2020	n/a	TNFα, CRP, IL: 1β, 6, 8	Right unilateral/bilateral; 3 ​× ​1 week	n/a	∼
	Lehtimäki et al., 2008	TAU	IL: 1β, 1Ra, 6	Bilateral x 1-7	n/a	∼
	Rush et al., 2016	TAU	TNFα, CRP, IL: 6, 10	Bilateral; 2 ​× ​1 week	SAL	∼
	Mindt et al., 2020	TAU	TNFα, IFNα/γ, MIP1α/β, IP10, MCP1, IL: 1β, 1Ra, 6, 8, 10, 12, 13,	Unilateral; 2/3 per week x 1–7 weeks	n/a	∼
	Belge et al., 2020	TAU	TNFα, IL6	Right unilateral; 2xweek x 2 weeks	n/a	∼
	Kargar et al., 2014	Celecoxib	TNFα, CRP, IL: 1, 6	Bilateral; 3 × week	SAL	AI
	Kartalci et al., 2016	Clozapine, haloperidol	IL4	Bilateral; 3 ​× ​3 weeks	n/a	∼
**L/Q** ∗	Gao et al., 2018	TAU	CRP, IL: 6, 10	Li: 300–600/Quet: 50–300 ​mg/day x 16 weeks	Li/Quet	∼
	Li et al., 2015	n/a	TNFα, IL: 10, 17, 23	Comb: Quet: 600–750 ​mg ​+ ​Li ​≥ ​0.6 ​mmol/L x 8 weeks	n/a	∼
**Quet.**	Fiedorowicz et al., 2019	IPSRT	CRP, IFNγ, IL: 4, 6, 10	50–300 ​mg/day x 20 weeks	Placebo	∼
	Igue et al., 2011	TAU	sIL2R, IL: 1Ra, 6	200–800 ​mg ​× ​12 weeks	n/a	∼
	Kao et al., 2016	n/a	TNFα, IFNγ, IL: 6, 10	400–800 ​mg/kg x 3 weeks	n/a	∼
**Li.**	Ricken et al., 2018	TAU	TNFα, IFNγ, IL: 6, 8, 10	0.715 ​mmol/l, duration NR	n/a	∼
	Merendino et al., 1994	NR	TNFα, IL6	900/1800 ​mg/day x 7 days	n/a	∼
**Ari.**	Juncal-Ruiz et al., 2018	TAU	TNFα, IFNγ, MIP: 1α/1β, 3α, IL: 1β, 2 6, 8, 10, 12, 13	5–30 ​mg/day x 3 months	Risperidone	∼
	Sobiś et al., 2015	Lorazepam	CRP, TNFα, IFNγ, IL: 1β, 1Ra, 6, 10, 12	10–30 ​mg/day x 4 weeks	n/a	∼
**Bup**	Tafseer et al., 2021	NR	TNFα	150 ​mg/day x 12 weeks	n/a	∼
	Eller et al., 2009	Escitalopram	sIL2R, IL8, TNFα	150–300 ​mg/day x 6 weeks	n/a	∼

ECT ​= ​electroconvulsive therapy, Quet. ​= ​quetiapine, Li. ​= ​lithium, Ari. ​= ​aripiprazole, Bup. ​= ​bupropion, BD ​= ​bipolar disorder, MS ​= ​mood stabilizer, TAU ​= ​treatment as usual, SAL ​= ​saline, DM ​= ​dexmedetomidine, IPSRT ​= ​interpersonal & social rhythm therapy, n/a ​= ​not applicable, nr ​= ​not reported, IL= Interleukin, α ​= ​alpha, β ​= ​beta, γ ​= ​gamma, TNFα ​= ​tumor necrosis factor, IFN ​= ​interferon, CRP ​= ​c-reactive protein, MIP ​= ​macrophage inflammatory protein, MCP ​= ​monocyte chemoattractant protein, RA ​= ​receptor antagonist, sIL-2R ​= ​soluble IL-2 receptor, mg ​= ​milligram, IV ​= ​intravenous, PRO ​= ​propofol, PI ​= ​potential pro-inflammatory effect of factor other than treatment of focus, AI ​= ​potential anti-inflammatory effect of factor other than treatment of focus.

**Fig. 2:**
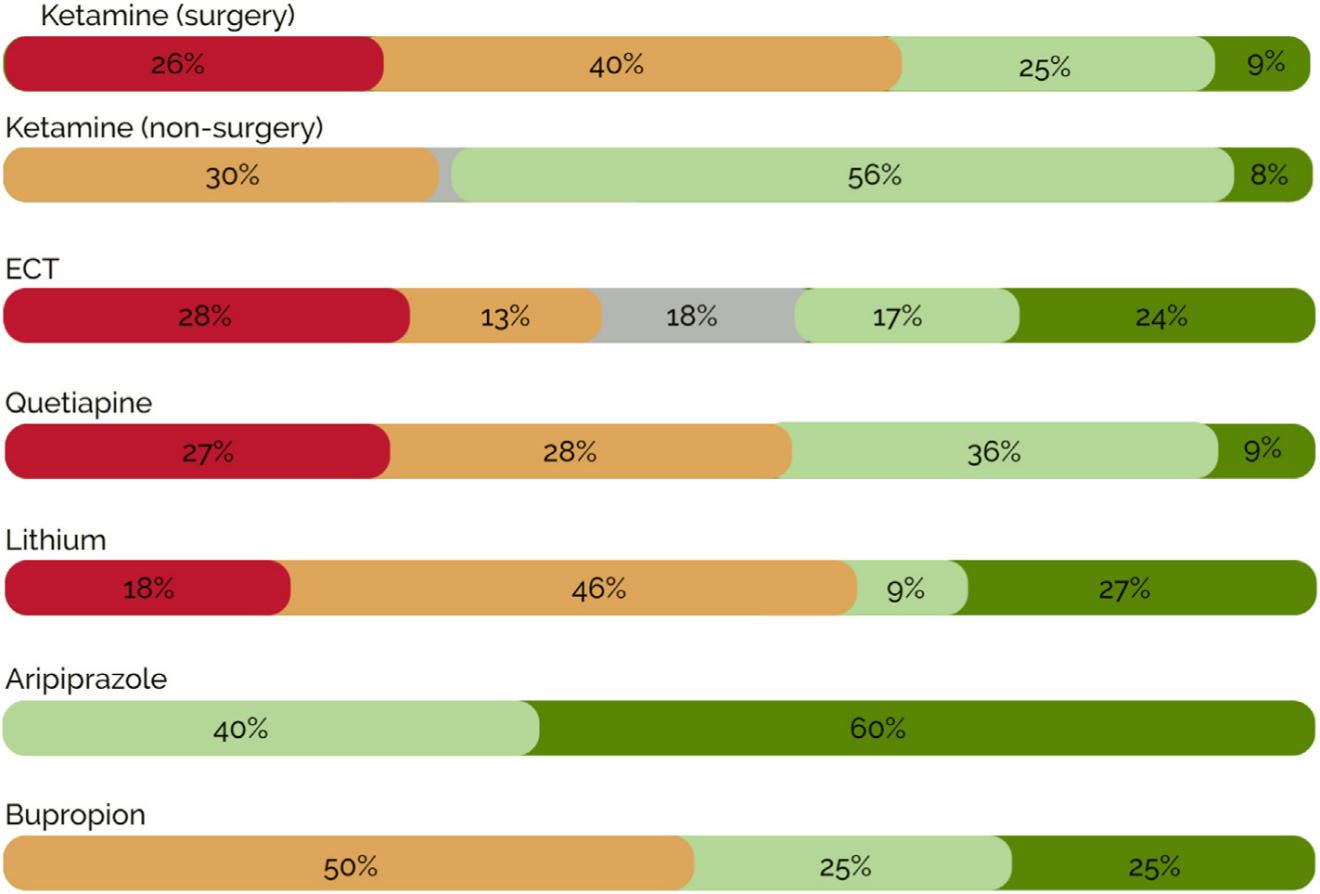
Summary of pro-inflammatory changes across treatments. Colour coding represents the strength and direction of pro-inflammatory effects: red ​= ​significant increase, orange ​= ​non-significant increase, grey ​= ​no change, light green ​= ​non-significant decrease, dark green ​= ​significant decrease. The % represent the proportion of all pro-inflammatory effects categorised according to each colour across studies for each treatment. Note that for the treatments towards the bottom, these rates are based on few comparisons. (For interpretation of the references to colour in this figure legend, the reader is referred to the Web version of this article.)

Inflammatory effects of treatments are initially described through summarising the direction and significance of changes in assessed biomarkers across all studies for each treatment. These are summarised by ‘comparisons’ or ‘assessments’ (i.e., all biomarker comparisons across all studies summed together), reported using percentages below. This approach should be interpreted cautiously, since a simple dichotomisation between rises and falls of biomarker levels is somewhat reductionist, and in particular does not account for magnitude of effect and the influence of effect modifying factors. The majority of studies assessed pro-inflammatory markers (comprising 70–100% of assessments per treatment) and these are therefore initially focused on, as depicted in Fig. 2.

For ketamine, 66% of assessments indicated increases in pro-inflammatory markers (significant or non-significant), compared with 34% decreases in pro-inflammatory markers. For ECT, pro-inflammatory biomarker decreases (41%) were equally as likely as pro-inflammatory increases (41%). For quetiapine, pro-inflammatory increases were more likely (55%) than decreases (45%), with the rate of significant effects being lower than other treatments (27% significant increases, 9% significant decreases). For lithium (despite sharing some of the same datapoints from the one trial treating participants concomitantly with lithium and quetiapine), increased pro-inflammatory markers were more common (64%) than decreases (36%), with few significant effects, these being slightly more frequent for decreases (27%) than increases (18%). Across 2 aripiprazole studies, all 15 pro-inflammatory marker comparisons showed significant (60%) or non-significant (40%) reductions after treatment. Finally, the two bupropion studies (with only four pro-inflammatory cytokine comparisons) reported equal frequency of increases and decreases during treatment.

A more detailed summary of these findings is presented in Table 3; this shows, for each treatment, the number of studies in which each biomarker was measured, then broken down into the number where significant/non-significant increases and decreases were reported during treatment, as a summary of intervention effects on each marker. The full results for all included studies can be found in Supplementary Table 1 (A-F). Below we consider a summary of results for each treatment, reporting both the proportion of pro/anti-inflammatory comparisons (across biomarkers and studies) in terms of direction and significant, and the effects of potential effect modifiers that could be considered for each.

**Table 3:**
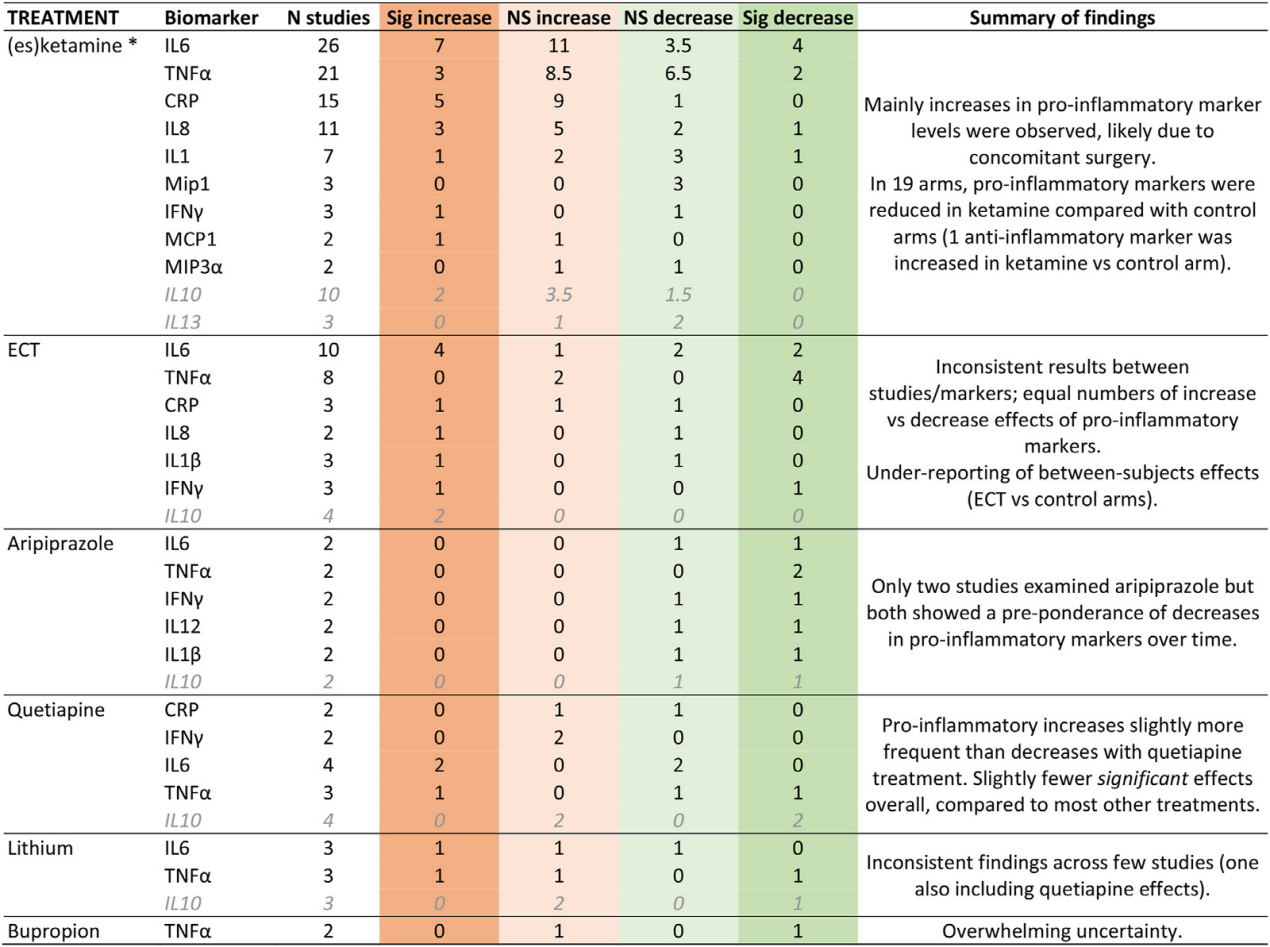
Summary of primary outcomes across treatments, studies and biomarkers.

#### 3.3.1 Ketamine

Many of the biomarker effects observed were non-significant; 33% were reported to be statistically significant within-subjects effects, 25% of which indicated pro-inflammatory increases and 8% decreases. Of the few anti-inflammatory marker comparisons (63% of which numerically increased), there were two reports of significant increases in levels while none showed statistically significant decreases.

*Surgery confounding:* 27/34 (79%) ketamine studies were likely confounded by surgical procedures that participants were undergoing between pre- and post-treatment biomarker assessments. In the remaining 7 studies, almost two thirds of comparisons indicated anti-inflammatory effects, although the majority were non-significant (Table 3).

*Timing after administration/surgery:* Post-ketamine biomarker assessments were undertaken at a range of timepoints following administration. Decreases in pro-inflammatory markers were short lasting: for those taken less than 12 ​h (range 30 ​min–4 ​h) afterwards, a preponderance of significant decreases in pro-inflammatory markers were observed (85% of comparisons), whereas those measured between 12 and 24 ​h were more often increased compared to baseline (84%). By 48 ​h, many were increased either non-significantly (56%) or significantly (28%). The one study with a longer interval (72 ​h) reported all non-significant changes compared to baseline.

*Anomalous studies:* Two of the ketamine studies were unusual in methodology, with one examining infants (Bhutta et al., 2012) and another being naturalistic in nature (Park et al., 2017). The infant study's results did not appear notably different from others, reporting all non-significant within-subjects effects (mixture of pro- and anti-inflammatory increases and decreases) 24 ​h after ketamine. The naturalistic (surgery) study examined two pro-inflammatory markers, reporting significant decreases in IL-6 (as another did 24 ​h after ketamine (Kartalov et al., 2012)) and non-significant increases in TNFα as several other studies did. The only high RoB study reported one significant and one non-significant inflammatory marker increase 24 ​h after surgery (Yang et al., 2006). Three surgery studies were of esketamine (all others were the racemic mixture ketamine); notably, two of three esketamine studies reported consistent and significant decreases in several pro-inflammatory cytokines (the only ketamine studies for which this was the case) (Ali and Mokhtar, 2017; Mostafa et al., 2008), while the other reported consistently non-significant (increase and decrease) effects (Silva et al., 2012).

*Between-group comparisons:* 19 post-treatment biomarker comparisons were reported to be significantly different between ketamine vs control treated participants, all with decreased pro-inflammatory levels in the ketamine group. All but one of these reports was from surgery studies. No studies reported higher pro-inflammatory marker levels in participants treated with ketamine vs control (usually saline). This suggests that the frequently acute pro-inflammatory increases observed with ketamine are most likely an artifact of various surgical procedures.

*Mood disorders samples:* Of the 7 non-surgical studies, 6 recruited patients with affective disorders (four TRD and two MDD). As the only non-surgical non-mood study only assessed one biomarker, these patients represent the majority of comparisons assessing ketamine without the likely confounding effect of surgery, which are more likely to indicate (largely non-significant) reductions in inflammatory activity after ketamine.

#### 3.3.2 ECT

Approximately half of the pro-inflammatory marker comparisons were reported to be statistically significant, with 28% indicating significant increases and 24% decreases. Of the 4 anti-inflammatory marker comparisons, 2 indicated significant increases and the other two showed no change.

*Timing after administration/surgery:* As with ketamine, timing of the post-treatment biomarker assessments after the procedure is likely to contribute to heterogeneity of findings. For four studies, this was 24 ​h afterwards [50% significant pro-inflammatory increases; 30% significant pro-inflammatory decreases), while two studies assessed this 2–6 days afterwards [2 significant pro-inflammatory increases, 2 significant pro-inflammatory decreases and no non-significant effects]. The 5 studies exceeding one week were evenly distributed across directions of effect, with most being non-significantly different from baseline.

*Duration of treatment:* This varied between studies (e.g., from two treatments over one week (Rush et al., 2016) to twice per week for 7 weeks (Fluitman et al., 2011). This also varied within studies, with many reporting a wide range between participants (e.g., between 6 and 12 sessions (Hestad et al., 2003)). The latter made it difficult to undertake an examination of treatment intensity, or duration, as a subgroup for results. However, the least intensive ECT overall (Rush et al., 2016) identified a significant increase in IL-6 48 ​h after the last session, and the most intensive ECT overall (Fluitman et al., 2011) reported significant decrease in IL-6 and TNFα, alongside a significant increase of IFNγ and IL-10 150 ​h after the last session.

*Anomalous studies:* Study design was fairly evenly distributed between randomised, non-randomised and naturalistic studies; due to the number of studies overall, this was therefore not examinable as a potential subgroup. One study administered concomitant celecoxib (an anti-inflammatory medication) (Kargar et al., 2014). This reported a significant decrease in TNFα (with non-significant decreases in IL-6 and CRP, and a non-significant increase in IL-1β) two weeks after ECT (three other studies also observed significant decreases in this pro-inflammatory cytokine, albeit closer to treatment end (Fluitman et al., 2011; Hestad et al., 2003; Zincir et al., 2016)). One other study was notably different from others regarding the population studied, treatment-resistant schizophrenia (Kartalci et al., 2016). This only assessed one anti-inflammatory marker (IL-4), finding a significant increase after ECT (similar to one of the other four anti-inflammatory comparisons for this treatment (IL-10 (Fluitman et al., 2011)).

*Mood disorders samples:* It is not unsurprising that all other ECT studies recruited participants with mood disorders, as this is the primary indication for this treatment. As shown in Table 1, the types of mood disorders varied across these 11 studies to the extent that it was not possible to differentiate findings based on specific population. The two TRD studies (Fluitman et al., 2011; Zincir et al., 2016) reported significant changes in markers, albeit measured at very different durations after ECT. Both reported reductions in TNFα and increases in IL-10, but significant findings in opposite directions were reported for IL-6 and IFNγ.

#### 3.3.3 Quetiapine

Across the five quetiapine studies, increases in pro-inflammatory markers were more frequent than decreases, but significant changes were relatively uncommon. For anti-inflammatory comparisons, IL-10 significantly decreased in two studies, and non-significantly increased in two (while a non-significant decrease was found for IL-1RA and an increase in IL-4, in single studies.)

*Treatment duration:* Duration of quetiapine treatment varied from 3 weeks (Kao et al., 2016) to 20 weeks (Fiedorowicz et al., 2019) (with 8, 12 and 16 weeks in between). This variation hindered our ability to explore duration effects on inflammation. However, the shortest study reported a mixture (significant and non-significant) of pro- and anti-inflammatory increases and decreases; conversely, the longest study reported increases consistently for both pro- and anti-inflammatory markers (Fiedorowicz et al., 2019).

*Anomalous studies:* Most notably, one of the five studies provided combined therapy with quetiapine and lithium (Li et al., 2015). This study reported more anti-inflammatory effects than the other quetiapine studies, which may be attributable to lithium treatment (although this is not reflected in between subjects findings, below, from the comparison study with lithium). One other study differed in its concomitant treatment which was with interpersonal and social rhythm (psycho)therapy (IPSRT); this was also the longest study (findings summarised above) with overall increases in cytokine levels (Fiedorowicz et al., 2019).

*Between-group comparisons:* The study administering concomitant IPSRT (both groups) compared quetiapine with placebo (Fiedorowicz et al., 2019), and found greater increases in pro-inflammatory markers (IL-6, TNFα) in quetiapine-treated patients. The only other study with a comparator arm compared quetiapine with lithium (Gao et al., 2018), finding no statistical difference between groups although quetiapine resulted in non-significant decreases (in IL-6, CRP; non-significant increase in IL-10) while the lithium group showed non-significant increase in all three markers.

*Mood disorders samples:* Three of the five studies included participants with bipolar disorder (two depressed, one manic) and the other two studies examined people with psychoses. The only notable difference was in the mania study (greater anti-inflammatory effect), which was the one administering both lithium and quetiapine; therefore, it is not possible to attribute this difference conclusively to either a population/symptom effect or a treatment effect.

#### 3.3.4 Lithium

Although the number of pro-inflammatory proteins increasing numerically was higher than the number reducing during lithium treatment, slightly more of the pro-inflammatory decreases were significant than for increases. With the three anti-inflammatory comparisons (all IL-10), two non-significantly increased and one significantly decreased.

*Treatment duration:* Duration of lithium treatment varied from 7 days (breast cancer study; see below) (Merendino et al., 1994), to 16 weeks (Gao et al., 2018), with another being 8 weeks (Li et al., 2015) and the other not reported (Ricken et al., 2018). We were therefore not able to examine the effect of lithium duration on inflammatory effects.

*Anomalous studies:* The study of lithium in people with breast cancer found both pro-inflammatory markers to increase significantly in the seven days of treatment (representing the only significant increases observed with lithium) (Merendino et al., 1994). The study treating participants with combined lithium and quetiapine is described above.

*Between-group comparisons:* Other than findings noted above comparing lithium and quetiapine, no lithium studies included a comparator treatment.

*Mood disorders samples:* The two studies examining lithium and quetiapine were for people with bipolar disorders, and the study not mentioned in detail was for people with MDD; this reported primarily non-significant increases in pro-inflammatory (IL-8, TNFα, IFNγ) and anti-inflammatory (IL-10) markers, the only exception being a minimal non-significant decrease in IL-6 (Ricken et al., 2018).

#### 3.3.5 Aripiprazole

Compared to other treatments, results for this antipsychotic were more likely to be significant and were the most consistent in terms of direction (indicating inflammatory reductions across markers in both studies). Its effects on anti-inflammatory cytokines were less consistent. Indeed, the only protein (of 19 comparisons) to increase over time was IL-10 in one study (the other 3 anti-inflammatory marker reductions were non-significant (IL-10) or significant (two studies, IL-13).

*Treatment duration:* One study assessed participants after 3 months of aripiprazole therapy (5–30 ​mg) (Juncal-Ruiz et al., 2018), and the other for 4 weeks (10–30 ​mg) (Sobiś et al., 2015), with relative consistency of inflammatory effects between studies.

*Anomalous studies:* In one study, participants with chronic schizophrenia had been switched to aripiprazole from standard atypical antipsychotics and the anti-inflammatory effects of aripiprazole were reportedly linked with weight loss in addition to clinical improvement. These anti-inflammatory effects may have represented a normalisation of inflammatory activity after increases with previous treatments that are known to produce metabolic syndromes (Sobiś et al., 2015). The other study differed in participants being in their first episode of psychosis and antipsychotic naïve (Juncal-Ruiz et al., 2018).

*Between-group comparisons:* The first-episode psychosis study compared aripiprazole with risperidone (also recommended for treatment-resistant depression but not strongly enough to have been pre-selected for systematic inclusion in this review); no statistical differences in inflammatory effects were reported between these two treatments but aripiprazole appeared to have greater overall anti-inflammatory effects (Juncal-Ruiz et al., 2018). The other study did not include a comparison treatment group.

#### 3.3.6 Bupropion

Of the four cytokine comparisons, one significant increase (in IL-8) (Eller et al., 2009) and one significant decrease (TNFα) (Tafseer et al., 2021) was reported (two non-significant). Three of the four comparisons were from the former study, which assessed a slightly shorter duration of bupropion treatment (6 vs 12 weeks) and where bupropion was augmented onto escitalopram (concomitant treatment not reported in the other study). The other notable difference between these studies was the latter recruiting participants with early stage TRD as opposed to MDD. It was not possible to ascertain the effects of these factors on inflammatory effects.

### 3.4 Risk of bias assessment

Risk of bias (ROB) ratings can be found in Supplementary Table 2. Of the 57 studies, 24 were rated as having a low ROB, 26 moderate ROB and 1 high ROB. Unclear ROB was categorised frequently, since it was often difficult to identify how the participants were assigned, allocation concealment, and if participants and those delivering the intervention were blinded to assigned intervention due to limited information reporting. The domain rated as the lowest risk of bias was appropriate outcomes being analysed (0% high ROB) and the domain rated as the highest risk of bias was potential for confounding after baseline to affect outcomes (52%). This was particularly the case for ketamine studies where generally participants were concomitantly undergoing medical surgery. As a result, ketamine studies were much less likely to be rated as having an overall low ROB.

## 4 Discussion

This systematic review aimed to determine the effects of TRD-recommended treatments on circulating inflammatory markers, across study and population types. Of the six treatments focused on, ketamine was the most studied, comprising over half of all included studies. ECT was also well represented, but there remains substantial uncertainty regarding the effects of lithium and quetiapine (with only 3–4 studies assessing both medications individually), as well as aripiprazole and bupropion (both two studies, including a total of <100 participants each). In many respects, the evidence for ECT is strongest, having the most studies with mood disorders and without clear non-treatment confounding influences on inflammatory markers (unlike the ketamine studies).

More increases than decreases (numerically) in pro-inflammatory markers were reported for ketamine and quetiapine. As summarised in Fig. 2, this comprised 55% for quetiapine and 66% of pro-inflammatory marker comparisons in ketamine surgery studies. However, non-surgery studies frequently reported non-significant reductions after ketamine (66% of pro-inflammatory marker comparisons), and esketamine may exhibit more anti-inflammatory effects than the racemic mixture. Numerically, increases were more frequent than decreases in inflammation for lithium (64% of pro-inflammatory comparisons), but significant effects were more likely to show decreased than increased pro-inflammatory cytokine levels. Increased/decreased pro-inflammatory markers were equally likely as one another for ECT and bupropion, while aripiprazole elicited consistent reductions in pro-inflammatory markers.

### 4.1 Results in context

Most treatments for depression do not work as well for patients who have elevated peripheral pro-inflammatory protein markers (Liu et al., 2019; Strawbridge et al., 2015, 2019a, 2020). Suggestions that anti-inflammatory treatments can alleviate depression (Jones et al., 2020; Nettis et al., 2021) led to a systematic review examining whether people with dysregulated inflammation may respond better to treatments classified as anti-inflammatory (Yang et al., 2019): This previous review concluded that for interventions with a known downregulatory effect on inflammation, people with high baseline pro-inflammatory markers responded better than those without, but for other treatments, there was either no predictive signal or high inflammation predicted a poorer treatment response. The treatments categorised as ‘anti-inflammatory’ in this previous review were ECT and ketamine (amongst others not typically prescribed for depression), while lamotrigine and SSRI/SNRIs were classed as not having a specific inflammatory action. The review did not, however, include specific data or substantive justification for classifying the treatments as such, which was one of the motivations for undertaking our review (Yang et al., 2019). Our findings do not support ketamine or ECT as consistently anti-inflammatory and are considered further below.

### 4.2 Ketamine

Ketamine is thought to exert anti-inflammatory effects at different levels, inhibiting cytokine production from mechanisms such as deactivating microglial cells and increased tryptophan metabolism (through quinolinic acid inhibition and subsequent metabolite modulation along the kynurenine pathway) (Kopra et al., 2021). In this context, the paucity of significant decreases in inflammatory markers in the reviewed studies is surprising given the accumulating evidence suggesting anti-inflammatory mechanisms of ketamine. The evidence appears particularly strong in preclinical studies, but recent reviews have concluded that despite heterogeneity in human studies, ketamine downregulates inflammation in at least a subset of depressed individuals (Kopra et al., 2021). An older meta-analysis also demonstrated significantly reduced IL-6 after surgical use of ketamine compared with control conditions (Dale et al., 2012). It is notable that ketamine use as an anaesthetic i.e., in high doses, may elicit distinct pharmacodynamic effects compared to sub-anaesthetic doses (Wu et al., 2020). The 7 non-surgery studies that we reviewed support the possibility of anti-inflammatory effects to some extent, although even after removing the significant confound of surgery, most cytokine reductions were non-significant. These were also mostly mood disorders patients, in contrast to surgery studies. Timing of the post-treatment inflammation assessment likely contributed to heterogeneity. Interestingly, though, the studies assessing biomarkers a short time after ketamine tended to show reductions; slightly later assessments indicated a normalisation, although around two days afterwards increased inflammation was more likely, thereafter appearing to normalise again. As previously, between-subjects effects (surgery with vs without ketamine) might be more relevant here, and these tended to show lower pro-inflammatory markers in ketamine-treated individuals (Dale et al., 2012). It is worth noting that most studies used the racemic mixture ketamine, but of only three esketamine studies, anti-inflammatory effects appeared more likely.

### 4.3 ECT

Similarly, previous studies have suggested anti-inflammatory mechanisms of ECT, which our findings do not necessarily support. Two recent meta-analyses suggested that initial acute increases in pro-inflammatory cytokines (particularly after initial sessions) are followed by reductions in the longer term (Gay et al., 2021; Desfossés et al., 2021) although one meta-analysis acknowledged a lack of *systematic* differences (Desfossés et al., 2021). The mechanism of anti-inflammatory action here may be secondary to neurotrophic effects on neurogenesis and synaptogenesis, particularly para-hippocampally (Desfossés et al., 2021). Again, timing of measurement after the procedure is likely to play a role; we identified initial increases in inflammation (aligning with previous evidence), followed by inconsistent directions of change (often significant in magnitude) in the days following and thereafter a normalisation. It is possible that more intensive ECT has greater anti-inflammatory effects in the days following the last session (Fluitman et al., 2011).

### 4.4 Quetiapine

There is less existing evidence on quetiapine's inflammatory effects, although previous evidence has suggested that quetiapine may reduce inflammation by inhibiting microglial activation (Bian et al., 2008). Other theories posit that antipsychotics associated with weight gain do so by suppressing pro-inflammatory, and raising anti-inflammatory, cytokines. In this respect, quetiapine may be expected to follow this pattern to a moderate degree (e.g., less than olanzapine but more than aripiprazole) (Fonseka et al., 2016). Although our findings are inconsistent (for both pro- and anti-inflammatory cytokines), increases in inflammation were indicated from the longest study which treated participants concomitantly with IPSRT. Although all markers showed increases in the latter study, the two significant rises (IL-6, TNF) were increased compared to participants under IPSRT ​+ ​placebo (and it is notable here that IPSRT could be expected to reduce inflammation via normalising circadian rhythms).

### 4.5 Lithium

Several reports strongly argue for lithium being substantively anti-inflammatory, largely via its inhibition of glycogen synthase kinase-3 (GSK3) (Beurel and Jope, 2014), and this has been supported in animal studies (Adams et al., 2020). Other reports, though, have argued that lithium is anti-inflammatory under some conditions and pro-inflammatory under others, with the specific circumstances for each remaining unestablished (Nassar and Azab, 2014). The latter is reminiscent of our findings. Our included study treating participants with concomitant lithium and quetiapine indicated more anti-inflammatory effects than other studies, whereas the study *comparing* lithium and quetiapine showed non-significant differences between them, albeit with lithium being less anti-inflammatory. However, where included studies reported *significant* effects of changes under lithium, these were more likely to indicate reductions in inflammation. In summary, a lack of clarity is apparent.

### 4.6 Aripiprazole

Our consistent results of inflammatory reductions after aripiprazole align with evidence that this has more protective effects on inflammation than other agents (Crespo-Facorro et al., 2021), including specifically atypical antipsychotics (Sobiś et al., 2015). However, this more consistent pattern of findings is perhaps surprising when considering the lack of consistency in the other reviewed treatments, many of which have more compelling evidence surrounding underlying anti-inflammatory mechanisms. It also contrasts with reports that antipsychotics associated with weight gain have more significant effects on inflammation (Fonseka et al., 2016). The small number of studies and participants, however, limits the conclusions that can be drawn from this.

### 4.7 Bupropion

Bupropion has, in few reports, been heralded for its potential anti-inflammatory effects. These range from animal studies (Brustolim et al., 2006), commentaries (proposing a contrast with other antidepressants such as mirtazapine) (Kast, 2003) and case studies of efficacy in Crohn's (Kast and Altschuler, 2001). However, other animal studies have found more inconsistent effects (Hajhashemi and Khanjani, 2014), which is similar to the limited evidence base that we have found in humans.

### 4.8 Heterogeneity

We highlight a distinct lack of homogeneous findings, which undoubtably reflects the variable methodologies of included studies. This is a strength and a weakness of our review; broad inclusion criteria have permitted an incorporation of all possible human evidence but this inevitably leads to complexity in synthesising findings throughout the literature. Heterogeneity is evident in population (few mood disorders studies) and in design (with a mixture of controlled interventional and naturalistic studies). Both of these factors were considered relatively unlikely to influence our primary outcome of inflammatory changes after treatment, although these were considered as subgroups in results where feasible. A potentially more important source of heterogeneity is treatment intensity (in dose, frequency and duration), which may differentially impact inflammatory activity between interventions. Another is concomitant treatment, which in many studies was ‘usual care’, something varying even within study samples and likely to influence cytokine changes. Thirdly, as has already been discussed, the timing of inflammatory marker assessments after treatments (in minutes, hours or days). Our findings may have differed somewhat had we selected a different priority order for selecting the post-treatment outcome timepoint, where multiple endpoints were reported (for ECT or ketamine).

The fluctuating nature of these biomarkers contributes in all studies to heterogeneity of results. Inflammatory proteins have a relatively low stability over time and are influenced by numerous factors including stress, diet, exercise, substance use and even the conditions of sample storage after blood collection (Strawbridge et al., 2018b). Some, but not all, relevant factors were adjusted for in some studies, captured by our risk of bias assessment. A refined modelling of these effects is not possible, and the uncontrolled studies of a longer duration may have been particularly vulnerable in this respect. Also relevant here is heterogeneity between participants within studies, which will have varied according to many of the above factors as well as baseline inflammatory states and continuation treatments, among other variables.

### 4.9 Strengths and limitations

Some strengths and limitations are noted above, including the review's inclusive approach to study selection, heterogeneity and small evidence bases. The size (as well as number) of included studies is also important to highlight; almost all studies were underpowered, with only 4 studies including ≥100 participants (3 intraoperative ketamine (Altiparmak et al., 2018; Cho et al., 2021; Welters et al., 2011; Ma et al., 2013) and the short-term lithium breast cancer study (Merendino et al., 1994)). Each of these four suffer other issues related to confounding or treatment intensity with none including people with mood disorders.

In aiming to undertake a relatively comprehensive but broad review, we did not consider *all* treatments used for TRD. Other possible interventions are generally not classified as anti-inflammatory and the metabolic side effects of some (e.g., olanzapine, risperidone) may reduce their appropriateness for people with inflammatory depression (Fonseka et al., 2016). Specifically, in the most recent meta-analysis of efficacy data from RCTs of augmentation trials for TRD, the only pharmacological interventions trialled in ≥4 studies were those included in this review (4–13 studies, totalling over 400 participants) in addition to brexpiprazole (low effect size), risperidone and thyroid hormones (few participants; <300) (Scott et al. 2022). Although we could have examined additional treatments, our selection was made prior to the start of the review process and published in our protocol for transparency.

A critical point is that our approach to synthesising inflammatory outcomes was somewhat reductionist, both in dichotomising these biomarkers as simplistically ‘pro’ or ‘anti’ inflammatory (Strawbridge et al., 2018b) and in assessing the importance of the increases or decreases for each intervention as significant or non-significant. We were also not able to comprehensively review the anti-inflammatory cytokine effects, which were assessed and reported rarely in comparison to pro-inflammatory cytokines. These limitations could have been addressed using a quantitative meta-analysis, had the quantity of evidence been sufficient to undertake meaningful meta-regressions or subgroup analyses.

It is possible that some relevant studies were omitted by our search strategy despite the systematic literature search including handsearching. A quantitative meta-analysis was not considered appropriate, due to the extensive heterogeneity between studies in terms of biomarker, participants, treatment and methodological characteristics, although it is possible that doing so may have indicated significant effects overall, in some cases.

### 4.10 Can inflammatory markers be used to improve treatment selection for TRD?

In a stratified model for TRD, patients with certain characteristics (in this case, an aberrant inflammatory profile) would be directed to treatments with more pronounced anti-inflammatory actions, while those without would be directed to other effective treatments. Previous reviews indicate that this could be fruitful (Yang et al., 2019), but ours does not bolster attempts to classify which of these treatments are ‘anti-inflammatory’. We foresee several routes through which inflammatory biomarkers could be used to improve outcomes for people with TRD: one would be a concerted effort to better delineate the effects of key treatments on inflammatory activity, which we have found lacking to date. This would strengthen possible future stratified treatment models. Second, a stratified trial could be undertaken without the above evidence gathering, on a more speculative than evidence-based foundation, although supported by previous work (Yang et al., 2019). Here, individuals with MDD/TRD would be randomised to a standard treatment or personalised treatment (e.g., anti-inflammatory if high pro-inflammatory markers) to ascertain whether the latter enhances response. Finally, evidence could focus on augmenting usual TRD treatments with specific anti-inflammatory medications for patients with elevated pro-inflammatory markers. This has previously been considered promising (Nettis et al., 2021) but to date not yielded consistent benefits (Husain et al., 2020).

In the current review, however, we have not identified robust evidence of either the nature or extent of peripheral inflammatory effects following existing TRD interventions. All of the reviewed interventions are worthy of further investigation. This is particularly the case for those that have long been considered to have downregulatory effects on inflammation (lithium, ketamine, ECT). However, we also urge future examination of the treatments with the most uncertainty but exhibiting potential, either given previous early indications (bupropion) or the more consistent findings we report here (aripiprazole), albeit based on a small number of studies.

## Declaration of competing interest

In the last 3 years: RS declares an honorarium from Lundbeck and Janssen. AHY declares honoraria for speaking from Astra Zeneca, Lundbeck, Eli Lilly, Sunovion; honoraria for consulting from Allergan, Livanova and Lundbeck, Sunovion, Janssen; and research grant support from Janssen. AJC has received honoraria for speaking from Lundbeck; honoraria for consulting from Allergan, Livanova, Janssen, and Lundbeck; and sponsorship for attending an academic conference from Janssen.
