# Supplementary material for: Peripheral inflammatory effects of different interventions for treatment-resistant depression: A systematic review
Source: Neurosci Appl. 2022 Nov 1;2:101014. doi: 10.1016/j.nsa.2022.101014 (PMC12244173; doi:10.1016/j.nsa.2022.101014)
Supplement: Multimedia component 1 [file mmc1.docx]

**Supplementary material**

**Supplement 1A:** Results of the effect of (es)ketamine on inflammatory markers

| **Study** | **Biomarker** | **Pre-treatment level *** | **Post-treatment level *** | **Post-treatment time** | **Longitudinal changes** |
| --- | --- | --- | --- | --- | --- |
| Akhlagh et al., 2010 | CRP | 10 (17) mg/dl | 23 (10) mg/dl | 48 hours | NS increase |
| Ali et al., 2017 | TNFα | 2053 (59) u/ml | 1253 (71) u/ml | 4 hours | Sig decrease (+ vs ctrl) |
|  |  | 2001 (30) u/ml | 906 (36) u/ml | 4 hours | Sig decrease (+ vs ctrl) |
|  | IL6 | 1089 (86) | 699 (10) | 4 hours | Sig decrease (+ vs ctrl) |
|  |  | 1150 (66) | 413 (16) | 4 hours | Sig decrease (+ vs ctrl) |
| Altiparmak et al., 2018 | CRP | 3 (1) mg/L | 42 (9) mg/L | 24 hours | Sig increase, but less than ctrl |
| Silva et al., 2012 | IL6 | 7 (13) | 31 (27) | 24 hours | NS increase |
|  |  | 7 (11) | 33 (18) | 24 hours | NS increase |
|  | *IL10* | 32 (59) | 350 (47) | 24 hours | NS increase |
|  |  | 86 (151) | 74 (145) | 24 hours | NS decrease |
|  | TNFα | 106 (95) | 75 (73) | 24 hours | NS decrease |
|  |  | 199 (388) | 155 (379) | 24 hours | NS decrease |
| Bhutta et al., 2012 | CRP | ~0.1 pg/L | 62 (28) pg/L | 48 hours | Sig increase, but less than ctrl |
|  | IL1α | 2.2 (0.6) | 2.1 (0.7) | 48 hours | NS decrease |
|  | *IL1Ra* | 2.1 (0.4) | 2.4 (0.4) | 48 hours | NS increase |
|  | IL6 | 1.5 (0.5) | 2.0 (0.2) | 48 hours | NS increase |
|  | IL8 | 1.2 (0.4) | 2.0 (0.2) | 48 hours | NS increase |
|  | *IL10* | 2.0 (0.4) | 1.7 (0.4) | 48 hours | NS decrease |
|  | *IL13* | 2.0 (0.5) | 2.1 (0.5) | 48 hours | NS increase |
|  | MIP1α | 1.2 (0.2) | 1.1 (0.4) | 48 hours | NS decrease |
|  | MCP1 | 1.6 (0.4) | 1.8 (0.2) | 48 hours | NS increase |
| Chen et al., 2018 | CRP | ~2.99 | ~3.05 | 72 hours | NS increase |
|  |  | ~2.83 | ~2.88 | 72 hours | NS increase |
|  | IL6 | ~4.46 | ~4.45 | 72 hours | NS decrease |
|  |  | ~4.44 | ~4.45 | 72 hours | NS increase |
|  | TNFα | ~2.89 | ~2.86 | 72 hours | NS decrease |
|  |  | ~2.85 | ~2.86 | 72 hours | NS increase |
| Cho et al., 2009 | CRP | ~1 mg/dl | ~16 mg/dl | 48 hours | Sig increase |
|  | IL6 | ~0.4 mg/dl | ~80 mg/dl | 48 hours | NS increase |
|  | TNFα | ~1.6 mg/dl | ~1.75 mg/dl | 48 hours | NS increase |
| Cho et al., 2021 | IL6 | lg 0.1 (CI -0.1-0.30) | lg 1.2 (1.1-1.3) | 48 hours | Sig increase |
|  | TNFα | lg 0.2 (CI 0.1-0.2) | lg 0.3 (0.3-0.4) | 48 hours | Sig increase |
|  | CRP | lg .05 (CI -0.2-0.3) | lg 1.81 (1.73-1.9) | 3 days | NS increase |
| D’ Alonzo et al., 2011 | IL6 | ~30 | 245 (287) | 24 hours | Sig increase |
|  | CRP | ~1.8 mg/dl | 9 (5) mg/dl | 24 hours | NS increase |
| Du et al., 2011 | IL6 | ~1.5 | ~1.6 | 1 hour | NS increase, but less than ctrl |
|  | TNFα | ~4.5 | ~3.9 | 1 hour | NS decrease (& vs ctrl) |
| Fiorelli et al., 2015 | CRP | 1.1 (0.2) mg/dl | 7.7 (1.7) | 48 hours | NS increase, but less than ctrl |
| Huang et al., 2006 | TNFα | 86 (7) ng/L | 90 (7) ng/L | End of surgery | NS increase |
|  |  | 91 (4) ng/L | 88 (11) ng/L | End of surgery | NS decrease |
|  | IL6 | 683 (186) ng/L | 672 (166) ng/L | End of surgery | NS decrease (& vs ctrl) |
|  |  | 691 (211) ng/L | 668 (183) ng/L | End of surgery | NS decrease (& vs ctrl) |
|  | IL8 | 1176 (286) ng/L | 1092 (141) ng/L | End of surgery | NS decrease |
|  |  | 1134 (348) ng/L | 1071 (327) ng/L | End of surgery | NS decrease |
| Hudetz et al., 2009 | CRP | 0.7 (0.8) mg/dl | 8.3 (2.0) mg/dl | 24 hours | Increase, but less than ctrl |
| Ibrahim et al., 2017 | IL6 | 11 (3) | 258 (28) | 24 hours | Sig increase |
|  |  | 15 (5) | 258 (28) | 24 hours | Sig increase |
|  | IL8 | ~13 | ~30 | 24 hours | Sig increase |
|  |  | ~17 | ~48 | 24 hours | Sig increase |
|  | *IL10* | ~0.5 | ~0.8 | 24 hours | Sig increase |
|  |  | ~0.5 | ~0.9 | 24 hours | Sig increase |
|  | TNFα | ~1.38 | ~1.25 | 24 hours | NS decrease |
|  |  | ~1.35 | ~1.47 | 24 hours | NS increase |
|  | CRP | ~18 | ~79 | 24 hours | Sig increase |
|  |  | ~16 | ~86 | 24 hours | Sig increase |
| Kartalov et al., 2012 | IL1β | 1.1 (0.3) | 2.0 (3.4) | 24 hours | NS increase |
|  | TNFα | 0.17 (0.03) | 0.23 (0.17) | 24 hours | NS increase |
|  | IL6 | 1.7 (0.9) | 9.4 (7.9) | 24 hours | Sig decrease (& vs ctrl) |
| Kawaguchi et al., 2020 | TNFα | 1 (0.4) | 1.1 (0.4) | 24 hours | NS increase |
|  | IL6 | 1.3 (0.8) | 27.1 (13.5) | 24 hours | NS increase, but less than ctrl |
| Kiraly et al., 2017 | IL6 | 0.35 | 0.25 | 24 hours | NS decrease |
|  | IL1β | 0.35 | 0.30 | 24 hours | NS decrease |
|  | TNFα | 0.60 | 0.66 | 24 hours | NS increase |
| Luggya et al., 2017 | IL6 | 10 (167) | 50 (316) | 48 hours | NS increase |
| Ma et al., 2013 | IL6 | 29 (2) | 31 (2) | 24 hours | NS increase |
|  |  | 28 (1) | 28 (1) | 24 hours | ~ |
| Mkrtchian et al., 2020 | CRP | 1.51 (2.55) | 1.39 (2.02) | 24 hours | NS decrease |
| Mostafa et al., 2008 | IL6 | 1190 (50) | 550 (20) | 30 minutes | Sig decrease |
|  |  | 1180 (55) | 380 (30) | 30 minutes | Sig decrease |
|  | IL8 | 1502 (160) | 850 (50) | 30 minutes | Sig decrease |
|  |  | 1500 (167) | 600 (40) | 30 minutes | Sig decrease |
|  | TNFα | 2170 (160) u/ml | 1110 (180) u/ml | 30 minutes | Sig decrease |
|  |  | 2176 (170) u/ml | 1000 (180) u/ml | 30 minutes | Sig decrease |
| Park et al., 2017 | IL6 | 1.15 | 1.07 | 24 hours | Sig decrease |
|  |  | 1.35 | 1.30 | 24 hours | Sig decrease |
|  | TNFα | 1.58 | 1.62 | 24 hours | NS increase |
|  |  | 1.87 | 1.95 | 24 hours | NS increase |
| Roytblat et al., 1998 | IL6 | 6 (9) | 85 | 48 hours | Sig increase, but less than ctrl |
| Senapathi et al., 2016 | CRP | 7 (6) mg/L | 32 (30) mg/L | 24 hours | Sig increase, but less than ctrl |
| Senapathi et al., 2019 | *IL10* | 13 (3) | 48 (4) | 24 hours | Sig increase +vs ctrl |
|  | IL8 | 36 (5) | 44 (5) | 24 hours | NS increase |
|  | CRP | 2.7 (1.4) | 3.5 (1.3) | 24 hours | NS increase |
| Singh et al., 2020 | CRP | 2 (1) mg/dl | 67 (6) mg/dl | 48 hours | Increase, but less than ctrl |
|  | IL6 | 22 (4) mg/dl | 83 (13) mg/dl | 48 hours | Increase, but less than ctrl |
|  | TNFα | 12 (2) mg/dl | 5 (1) mg/dl | 48 hours | NS decrease |
| Tu et al., 2007 | IL6 | ~71 | ~560 | 18 hours | Sig increase |
|  | IL8 | ~13 | ~971 | 18 hours | Sig increase |
|  | *IL10* | ~2 | ~2 | 18 hours | ~ |
|  | IFNγ | ~3 | ~20 | 18 hours | Sig increase |
|  | TNFα | ~3 | ~21 | 18 hours | Sig increase |
|  | IL1α | ~5 | ~8 | 18 hours | Sig increase |
|  | IL1β | ~1 | ~13 | 18 hours | Sig increase |
|  | MCP1 | ~361 | ~1167 | 18 hours | Sig increase |
| Wang et al., 2017 | IL1β | 34 (6) | 13 (10) | NR | Sig decrease (+ vs ctrl) |
| Welters et al., 2011 | IL6 | ~5 | ~45 | 24 hours | [Increase] |
|  | *IL10* | ~5 | ~5 | 24 hours | ~ |
|  | TNFα | ~7 | ~7 | 24 hours | ~ |
|  | IL8 | ~1 | ~2 | 24 hours | [Increase] |
|  | CRP | 5.07 (2.5) | 102 (66) | 24 hours | [Increase] |
| Xie et al., 2015 | TNFα | 16 (5) ng/L | 19 (7) ng/L | 24 hours | Sig increase, but less than ctrl |
|  | IL6 | 13 (3) ng/L | 23 (7) ng/L | 24 hours | Sig increase, but less than ctrl |
|  | IL8 | 6.5 (1.8) ng/L | 7.4 (2.1) ng/L | 24 hours | Sig increase, but less than ctrl |
| Xin et al., 2007 | TNFα | 30 (13) ng/L | 33 (13) ng/L | 24 hours | NS increase |
|  | IL6 | 65 (20) ng/L | 69 (21) ng/L | 24 hours | NS increase |
|  | IL8 | 28 (12) ng/L | 33 (12) ng/L | 24 hours | NS increase |
|  | *IL10* | 36 (13) ng/L | 40 (13) ng/L | 24 hours | NS increase |
| Yang et al., 2006 | TNFα | 13 (7) | 16 (6) | 24 hours | NS increase |
|  | IL6 | 6 (4) | 15 (7) | 24 hours | Sig increase |
|  | *IL10* | 6 (7) | 21 (26) | 24 hours | NS increase |
| Zhan et al., 2020 | IFNγ | lg 0.99 (0.24) | lg 0.99 | 24 hours | ~ |
|  | *IL10* | lg 1.14 (0.50) | NR | 24 hours | NS increase |
|  | MIP3α | lg 1.08 (0.35) | NR | 24 hours | NS increase |
|  | *IL13* | lg 0.54 (0.38) | NR | 24 hours | NS decrease |
|  | IL1β | lg 0.09 (0.34) | NR | 24 hours | NS increase |
|  | IL6 | lg 0.22 (0.34) | NR | 24 hours | NS decrease |
|  | IL7 | lg 0.91 (0.34) | NR | 24 hours | NS increase |
|  | IL8 | lg 0.37 (0.26) | NR | 24 hours | NS increase |
|  | MIP1β | lg 1.14 (0.33) | NR | 24 hours | NS decrease |
|  | TNFα | lg 0.57 (0.17) | NR | 24 hours | NS decrease |
| Zhou et al., 2020 | *IL13* | 7 (11) | 6.4 (10) | 24 hours | NS decrease |
|  | *IL10* | 4.3 (1.9) | 4.3 (2.2) | 24 hours | ~ |
|  | IL6 | 20 (11) | 19 (12) | 24 hours | NS decrease |
|  | IL8 | 23 (26) | 16 (15) | 24 hours | NS decrease |
|  | MIP1β | 368 (433) | 308 (352) | 24 hours | NS decrease |
|  | MIP3α | 17 (14) | 16 (11) | 24 hours | NS decrease |
|  | IL1β | 2.0 (2.1) | 1.7 (1.7) | 24 hours | NS decrease |
|  | TNFα | 8.0 (9.4) | 6.8 (7.1) | 24 hours | NS decrease |
|  | IFNγ | 14 (12) | 12 (8) | 24 hours | NS decrease |

**Grey text indicates a definitive anti-inflammatory marker. Highlights denote the following: dark orange = significant increase; light orange = non-significant increase; light green = non-significant decrease; dark green = significant decrease (pro-inflammatory markers). Multiple rows per biomarker per study denote subgroups of participants.**

* biomarker levels reported as mean (SD) in pg/ml unless reported otherwise.

nr: not reported, NS = non-significant, IL= Interleukin, α = alpha, β = beta, TNFα= tumor necrosis factor, IFNγ= interferon gamma, CRP= c-reactive protein, MIP = macrophage inflammatory protein, MCP = monocyte chemoattractant protein, RA = receptor antagonist, sIL-2R= soluble IL-2 receptor, ctrl = control, sig = significant, pg = picogram, ml = millilitre, u = unit, mg = milligram, L = litre, dl = decilitre, ng = nanogram.

**Supplement 1B:** Results of electroconvulsive therapy (ECT) treatment in inflammatory markers

| **Study** | **Biomarker** | **Pre-treatment level *** | **Post-treatment level *** | **Post-treatment time** | **Longitudinal changes** |
| --- | --- | --- | --- | --- | --- |
| Belge et al., 2020 | IL6 | 0.86 (0.92) | 0.71 (1.03) | 1 week | NS decrease |
|  | TNFα | 2.26 (0.75) | 2.26 (0.94) | 1 week | ~ |
| Fluitman et al., 2011 | TNFα | 131 (73) | 118 (58) | 150 hours | Sig decrease |
|  | IL6 | 1758 (568) | 1626 (735) | 150 hours | Sig decrease |
|  | IFNγ | 14573 (16015) | 18958 (17772) | 150 hours | Sig increase |
|  | *IL10* | 250 (137) | 365 (235) | 150 hours | Sig increase |
| Hestad et al., 2003 | TNFα | 17 | 13 | 24 hours | Sig decrease |
| Jarventausta et al., 2017 | IL6 | 8 (5) | 5 (4) | 10 days | Sig decrease |
| Kargar et al., 2014 | TNFα | 15 (8) | 14 (7) | 2 weeks | Sig decrease |
|  | IL1β | 6 (5) | 5 (6) | 2 weeks | NS decrease |
|  | IL6 | 8 (23) | 10 (24) | 2 weeks | NS increase |
|  | CRP | 8 (15) | 4 (6) | 2 weeks | NS decrease |
| Kartalci et al., 2016 | *IL4* | 16 (6) | 19 (7) | 4 weeks | Sig increase |
| Kruse et al., 2018 | IL6 | 1.2 (1.4) | 3.4 (7.4) | 24 hours | Sig increase |
|  | IL8 | 3.5 (1.9) | 3.3 (1.6) | 24 hours | NS decrease |
|  | TNFα | 6.5 (2.8) | 6.7 (2.8) | 24 hours | NS increase |
|  | CRP | 2 (4) mg/L | 15 (21) mg/L | 24 hours | Sig increase |
| Kruse et al., 2020 | IL8 | 2.8 (1.9-3.4) | 2.9 (1.7-4.1) | 1 week | Sig increase |
|  | IL6 | 1.8 (1.3-2.6) | 1.5 (1.2-2.0) | 1 week | NS decrease |
|  | *IL-10* | 0.7 (0.5-1.0) | 0.7 (0.4-1.0) | 1 week | ~ |
|  | TNFα | 6 (5-8) | 7 (5-8) | 1 week | NS increase |
|  | CRP | 1 (0.3-3.0) | 2 (0.9-4.3) | 1 week | NS increase |
| Lehtimaki et al., 2008 | IL1β | ~0.07 | ~0.15 | 24 hours | Sig increase |
|  | IL6 | ~1.5 | ~3.0 | 24 hours | Sig increase |
| Mindt et al., 2020 | MCP1 | NR | NR | NR | NS increase |
|  | IL-2R | NR | NR | NR | NS increase |
|  | MIP1α | NR | NR | NR | NS increase |
|  | 21 other cytokines NS and direction not reported: IL: 1b, 1RA, 2, 4, 5, 6, 7, 10, 12, 13, 15, 17, TNFα, IFNa/y, GMCSF, MIP1b, IP10, MIG, Eotaxin, Rantes. | | | | |
| Rush et al., 2016 | IL6 | ~0.8 | ~0.96 | 48 hours | Sig increase |
| Zincir et al., 2016 | IL6 | 35 (37) | 45 (94) | 24 hours | Sig increase |
|  | TNFα | 86 (17) | 78 (11) | 24 hours | Sig decrease |
|  | *IL10* | 17 (25) | 32 (18) | 24 hours | Sig increase |
|  | IFNγ | 22 (21) | 12 (10) | 24 hours | Sig decrease |

**Grey text indicates a definitive anti-inflammatory marker. Highlights denote the following: dark orange = significant increase; light orange = non-significant increase; light green = non-significant decrease; dark green = significant decrease (pro-inflammatory markers). Multiple rows per biomarker per study denote subgroups of participants.**

* biomarker levels reported as mean (SD) in pg/ml unless reported otherwise.

nr: not reported, NS = non-significant, IL= Interleukin, α = alpha, β = beta, TNFα= tumor necrosis factor, IFNγ= interferon gamma, CRP= c-reactive protein, MIP = macrophage inflammatory protein, MCP = monocyte chemoattractant protein, sig = significant, pg = picogram, ml = millilitre, mg = milligram, L = litre, RA = receptor antagonist, IFN = interferon, GMCSF = granulocyte-macrophage colony-stimulating factor, IP10 = IFNγ induced protein, MIG = monokine induced by IFNγ.

**Supplement 1C:** Results of aripiprazole treatment in inflammatory markers

| **Study** | **Biomarker** | **Pre-treatment level *** | **Post-treatment level *** | **Duration on treatment** | **Longitudinal changes** |
| --- | --- | --- | --- | --- | --- |
| Juncal-Ruiz et al., 2018 | IL6 | 2.2 (1.9) | 2.0 (2.1) | 3 months | NS decrease |
|  | IL1β | 0.68 (0.95) | 0.55 (0.81) | 3 months | NS decrease |
|  | TNFα | 3.0 (1.6) | 2.4 (1.4) | 3 months | Sig decrease |
|  | IFNγ | 5.3 (4.1) | 4.7 (3.7) | 3 months | NS decrease |
|  | *IL10* | 4.7 (2.8)) | 2.0 (3.9) | 3 months | NS decrease |
|  | *IL13* | 4.1 (3.8) | 3.7 (3.9) | 3 months | Sig decrease |
|  | IL12 | 2.3 (1.2) | 2.1 (1.2) | 3 months | NS decrease |
|  | IL8 | 7.9 (8.8) | 3.8 (2.5) | 3 months | Sig decrease |
|  | MIP1α | 12 (7) | 9 (3) | 3 months | NS decrease |
|  | MIP1β | 10.4 (4.8) | 9.3 (4.2) | 3 months | Sig decrease |
|  | MIP3α | 4.0 (1.3) | 3.6 (1.2) | 3 months | NS decrease |
| Sobis et al., 2015 | IL1β | 8.9 (2.0) | 8.3 (2.1) | 28 days | Sig decrease |
|  | IL6 | 8.1 (1.4) | 7.0 (1.3) | 28 days | Sig decrease |
|  | TNFα | 8.2 (2.1) | 7.3 (1.8) | 28 days | Sig decrease |
|  | IFNγ | 32.8 (19.2) | 30.9 (17.4) | 28 days | Sig decrease |
|  | IL12 | 12. 8 (2.0) | 11.7 (1.9) | 28 days | Sig decrease |
|  | *IL1Ra* | 190.4 (6.9) | 182.8 (8.8) | 28 days | Sig decrease |
|  | *IL10* | 3.4 (0.5) | 3.9 (0.4) | 28 days | Sig increase |
|  | CRP | 4.1 (1.7) mg/L | 2.0 (0.9) mg/L | 21 days | Sig decrease |

**Grey text indicates a definitive anti-inflammatory marker. Highlights denote the following: dark orange = significant increase; light orange = non-significant increase; light green = non-significant decrease; dark green = significant decrease (pro-inflammatory markers). Multiple rows per biomarker per study denote subgroups of participants.**

* biomarker levels reported as mean (SD) in pg/ml unless reported otherwise.

nr: not reported, NS = non-significant, IL= Interleukin, α = alpha, β = beta, TNFα= tumor necrosis factor, IFNγ= interferon gamma, CRP= c-reactive protein, MIP = macrophage inflammatory protein, sig = significant, pg = picogram, ml = millilitre, mg = milligram, L = litre.

**Supplement 1D:** Results of quetiapine treatment in inflammatory markers

| **Study** | **Biomarker** | **Pre-treatment level *** | **Post-treatment level *** | **Duration on treatment** | **Longitudinal changes** |
| --- | --- | --- | --- | --- | --- |
| Fiedorowicz et al., 2019 | CRP | 2.9 (2.9) | 3.6 (2.8) | 20 weeks | [Increase] |
|  | IFNγ | 5 (3) | 9 (14) | 20 weeks | [Increase] |
|  | *IL4* | 0.06 (0.13) | 0.02 (0.01) | 20 weeks | [Increase] |
|  | IL6 | 0.55 (0.26) | 1.55 (1.56) | 20 weeks | Sig increase & vs ctrl |
|  | *IL10* | 0.33 (0.35) | 0.28 (0.16) | 20 weeks | [Increase] |
|  | TNFα | 2.6 (0.6) | 3.1 (0.7) | 20 weeks | Sig increase & vs ctrl |
| Gao et al., 2018 | IL6 | 2.3 (1.3) | 2.2 (1.1) | 16 weeks | NS decrease |
|  | *IL10* | 0.10 (0.10) | 0.18 (0.14) | 16 weeks | NS increase |
|  | CRP | 1.88 (2.74) | 1.36 (1.01) | 16 weeks | NS decrease |
| Igue et al., 2011 | IL6 | 1.4 (SE 0.2) | 1.2 (SE 0.2) | 12 weeks | NS decrease |
|  | *IL1RA* | 398 (SE 82) | 393 (SE 79) | 12 weeks | NS decrease |
|  | sIL2R | 133 (SE 23) | 193 (SE 24) | 12 weeks | Sig increase |
| Kao et al., 2016 | IL2 | 0.61 (0.84) | 0.85 (0.72) | 3 weeks | Sig increase |
|  | IL6 | 7.0 (6.9) | 6.5 (7.5) | 3 weeks | Sig increase |
|  | *IL10* | 2.9 (0.4) | 2.5 (0.5) | 3 weeks | Sig decrease |
|  | TNFα | 0.39 (0.74) | 0.22 (0.62) | 3 weeks | NS decrease |
|  | IFNγ | 1.62 (1.06) | 1.84 (0.84) | 3 weeks | NS increase |
| Li et al., 2015 – NB combination of lithium & quetiapine | TNFα | 62 (3) | 50 | 8 weeks | Sig decrease |
|  | IL23 | 71 (9) | 50 | 8 weeks | Sig decrease |
|  | IL17 | 13 (2) | 7 | 8 weeks | Sig decrease |
|  | *IL10* | 4.6 (2.1) | 3.5 | 8 weeks | Sig decrease |

**Grey text indicates a definitive anti-inflammatory marker. Highlights denote the following: dark orange = significant increase; light orange = non-significant increase; light green = non-significant decrease; dark green = significant decrease (pro-inflammatory markers). Multiple rows per biomarker per study denote subgroups of participants.**

* biomarker levels reported as mean (SD) in pg/ml unless reported otherwise.

nr: not reported, NS = non-significant, IL= Interleukin, α = alpha, β = beta, TNFα= tumor necrosis factor, IFNγ= interferon gamma, CRP= c-reactive protein, RA = receptor antagonist, sIL2R= soluble IL-2 receptor, ctrl = control, sig = significant, SE = standard error.

**Supplement 1E:** Results of lithium treatment in inflammatory markers

| **Study** | **Biomarker** | **Pre-treatment level *** | **Post-treatment level *** | **Duration on treatment** | **Longitudinal changes** |
| --- | --- | --- | --- | --- | --- |
| Gao et al., 2018 | IL6 | 2.8 (1.7) | 3.5 (2.6) | 16 weeks | NS increase |
|  | *IL10* | 0.10 (0.10) | 0.18 (0.14) | 16 weeks | NS increase |
|  | CRP | 0.96 (0.67) | 1.00 (0.80) | 16 weeks | NS increase |
| Merendino et al., 1994 | TNFα | 29 (9) | 604 (202) | 7 days | Sig increase |
|  |  | 80 (7) | 210 (60) | 7 days | Sig increase |
|  | IL6 | 4.3 (2.0) | NR | 7 days | Sig increase |
|  |  | 4.5 (1.4) | NR | 7 days | Sig increase |
| Ricken et al., 2018 | IL6 | 41.0 (63.1) | 40.6 (35.0) | 4 weeks | NS decrease |
|  | IL8 | 66 (34) | 92 (165) | 4 weeks | NS increase |
|  | *IL10* | 74.3 (51.9) | 75.6 (44.7) | 4 weeks | NS increase |
|  | TNFα | 26.1 (10.0) | 26.8 (8.8) | 4 weeks | NS increase |
|  | IFNγ | 26.5 (10.2) | 27.9 (13.2) | 4 weeks | NS increase |
| Li et al., 2015 – NB combination of lithium & quetiapine | TNFα | 62 (3) | 50 | 8 weeks | Sig decrease |
|  | IL23 | 71 (9) | 50 | 8 weeks | Sig decrease |
|  | IL17 | 13 (2) | 7 | 8 weeks | Sig decrease |
|  | *IL10* | 4.6 (2.1) | 3.5 | 8 weeks | Sig decrease |

**Grey text indicates a definitive anti-inflammatory marker. Highlights denote the following: dark orange = significant increase; light orange = non-significant increase; light green = non-significant decrease; dark green = significant decrease (pro-inflammatory markers). Multiple rows per biomarker per study denote subgroups of participants.**

* biomarker levels reported as mean (SD) in pg/ml unless reported otherwise.

nr: not reported, NS = non-significant, IL= Interleukin, α = alpha, β = beta, TNFα= tumor necrosis factor, IFNγ= interferon gamma, CRP= c-reactive protein, RA = receptor antagonist, sIL2R= soluble IL-2 receptor, ctrl = control, sig = significant, SE = standard error.

**Supplement 1F:** Results of bupropion treatment in inflammatory markers

| **Study** | **Biomarker** | **Pre-treatment level *** | **Post-treatment level *** | **Post-treatment time** | **Longitudinal changes** |
| --- | --- | --- | --- | --- | --- |
| Eller et al., 2009 | sIL2R | 568 (314) ku/L | 564 (216) ku/L | 6 weeks | NS decrease |
|  |  | 558 (247) ku/L | 535 (151) ku/L | 6 weeks | NS decrease |
|  | IL-8 | 6.5 (2.1) | 6.9 (2.3) | 6 weeks | Sig increase |
|  |  | 7.4 (3.4) | 7.6 (2.3) | 6 weeks | NS increase |
|  | TNFα | 6.6 (2.9) | 7.0 (2.9) | 6 weeks | NS increase |
|  |  | 6.4 (2.5) | 7.2 (2.4) | 6 weeks | NS increase |
| Tafseer et al., 2021 | TNFα | 4.5 (1.0) | 2.1 (0.8) | 12 weeks | Sig decrease |

**Grey text indicates a definitive anti-inflammatory marker. Highlights denote the following: dark orange = significant increase; light orange = non-significant increase; light green = non-significant increase; dark green = significant decrease (pro-inflammatory markers). Multiple rows per biomarker per study denote subgroups of participants.**

* biomarker levels reported as mean (SD) in pg/ml unless reported otherwise.

nr: not reported, NS = non-significant, IL= Interleukin, α = alpha, TNFα= tumor necrosis factor, sIL2R= soluble IL-2 receptor, sig = significant, ku/L = kilo unit per litre.

Supplementary table 2: risk of bias ratings for each study

|  | **Author** | **1** | **2** | **3** | **4** | **5** | **6** | **7** | **8** | **9** | ***Overall risk*** |
| --- | --- | --- | --- | --- | --- | --- | --- | --- | --- | --- | --- |
| **(es)ketamine studies** | Chen et al., 2018 | ? | ? | + | - | ? | + | + | + | + | *Moderate* |
|  | Kiraly et al., 2017 | ? | ? | + | **-** | ? | **+** | **+** | **+** | **+** | *Moderate* |
|  | Mkrtchian et al., 2020 | ? | + | + | - | + | + | + | + | + | *Moderate* |
|  | Park et al., 2017 | NA | NA | + | - | ? | + | + | + | + | *Moderate* |
|  | Zhan et al., 2020 | NA | NA | + | - | ? | + | + | + | + | *Moderate* |
|  | Zhou et al., 2020 | NA | NA | + | - | ? | + | + | + | + | *Moderate* |
|  | Wang et al., 2017 | + | ? | + | ? | ? | + | + | + | + | *Low* |
|  | Akhlagh et al., 2010 | + | + | + | - | + | + | + | + | + | *Moderate* |
|  | Ali et al., 2017 | + | + | + | - | + | + | + | + | + | *Moderate* |
|  | Silva et al., 2012 | + | + | + | - | + | + | + | + | + | *Moderate* |
|  | Bhutta et al., 2012 | + | ? | + | - | ? | + | + | + | + | *Moderate* |
|  | Cho et al., 2009 | + | + | + | - | + | + | + | + | + | *Moderate* |
|  | Cho et al., 2021 | + | + | + | - | + | + | + | + | + | *Moderate* |
|  | D’Alonzo et al., 2011 | + | + | + | - | + | + | ? | + | + | *Moderate* |
|  | Du et al., 2011 | + | + | + | - | + | + | + | + | + | *Moderate* |
|  | Fiorelli et al., 2015 | + | + | + | - | + | + | + | + | + | *Moderate* |
|  | Huang et al., 2006 | ? | ? | + | + | ? | + | + | + | + | *Low* |
|  | Hudetz et al., 2009 | + | + | + | - | + | + | + | + | + | *Moderate* |
|  | Ibrahim et al., 2017 | + | + | + | - | - | + | + | + | + | *Moderate* |
|  | Kartalov et al., 2012 | ? | ? | + | - | - | + | + | + | + | *Moderate* |
|  | Kawaguchi et al., 2020 | + | ? | + | - | + | + | + | + | + | *Moderate* |
|  | Luggya et al., 2017 | + | + | + | - | - | + | + | + | + | *Moderate* |
|  | Ma et al., 2013 | + | ? | + | - | ? | + | + | + | ? | *Moderate* |
|  | Mostafa et al., 2008 | + | ? | + | + | - | + | + | + | + | *Moderate* |
|  | Roytblat et al., 1998 | + | ? | + | - | ? | + | + | + | + | *Moderate* |
|  | Senapathi et al., 2016 | + | + | + | - | - | + | + | + | + | *Moderate* |
|  | Senapathi et al., 2019 | + | + | + | + | - | + | + | + | + | *Moderate* |
|  | Singh et al., 2020 | + | + | + | - | - | + | + | + | + | *Moderate* |
|  | Tu et al., 2007 | ? | ? | + | + | - | + | + | + | + | *Moderate* |
|  | Welters et al., 2011 | + | + | + | - | ? | + | + | + | + | *Moderate* |
|  | Xie et al., 2015 | + | ? | + | - | ? | + | + | + | + | *Moderate* |
|  | Xin et al., 2007 | ? | ? | + | - | ? | + | + | + | + | *Moderate* |
|  | Yang et al., 2006 | ? | ? | - | - | ? | + | + | + | + | *High* |
|  | Altiparmak et al., 2018 | NA | NA | + | - | ? | + | + | + | + | *Moderate* |
| **ECT studies** | Zincir et al., 2016 | NA | NA | + | + | ? | + | + | + | + | *Low* |
|  | Fluitman et al., 2011 | NA | NA | + | + | ? | + | + | + | + | *Low* |
|  | Hestad et al., 2003 | ? | - | + | + | - | + | + | + | + | *Moderate* |
|  | Jarventausta et al., 2017 | NA | NA | + | + | ? | + | + | + | + | *Low* |
|  | Kruse et al., 2018 | NA | NA | + | + | ? | + | + | + | + | *Low* |
|  | Kruse et al., 2020 | NA | NA | + | + | ? | + | + | + | + | *Low* |
|  | Lehtimaki et al., 2008 | NA | NA | + | + | ? | + | + | + | + | *Low* |
|  | Rush et al., 2016 | NA | NA | + | + | ? | + | + | + | + | *Low* |
|  | Mindt et al., 2020 | NA | NA | + | + | ? | + | ? | + | + | *Low* |
|  | Belge et al., 2020 | NA | NA | + | + | ? | + | + | + | + | *Low* |
|  | Kargar et al., 2014 | + | + | + | + | + | + | + | + | + | *Low* |
|  | Kartalci et al., 2016 | ? | ? | + | + | ? | + | ? | + | + | *Low* |
| **L/Q** * | Gao et al., 2018 | ? | ? | + | + | ? | + | + | + | + | *Low* |
|  | Li et al., 2015 | NA | NA | + | + | ? | + | + | + | + | *Low* |
| **Quet.** | Fiedorowicz et al., 2019 | + | + | + | + | + | + | + | + | + | *Low* |
|  | Igue et al., 2011 | NA | NA | + | + | ? | + | + | + | + | *Low* |
|  | Kao et al., 2016 | NA | NA | + | + | ? | + | + | + | + | *Low* |
| **Li.** | Ricken et al., 2018 | NA | NA | + | + | ? | + | + | + | + | *Low* |
|  | Merendino et al., 1994 | ? | ? | + | + | ? | + | + | + | + | *Low* |
| **Ari** | Juncal-Ruiz et al., 2018 | NA | NA | + | ? | ? | + | + | + | + | *Low* |
|  | Sobis ́et al., 2015 | NA | NA | + | + | ? | + | + | + | + | *Low* |
| **Bup** | Eller et al., 2009 | ? | ? | + | + | ? | + | + | + | + | *Low* |
|  | Tafseer et al., 2021 | NA | NA | + | + | ? | + | ? | + | + | *Low* |

Item numbers correspond to the domains delineated below, alongside scoring criteria. NA = not applicable (due to study design); these ratings are conservatively treated as ‘uncertain’ in overall ROB ratings. **+ =** low risk/ **- =** high risk/ **?** = unclear risk

Overall risk was judged as follows: *Low risk = <1 criteria rated high RoB and <4 unclear RoB*

*High risk = >4 criteria rated high or unclear RoB*

*Moderate risk if not meeting criteria for high or low risk of bias.*

| **No.** | **Item** | **Scoring** |
| --- | --- | --- |
| **1** | **Random allocation**  Were the participants randomised to treatment in a truly random fashion? | + = yes (e.g. electronic random generator)  - = no (e.g. alternate)  ? = unclear or not reported. NA = if not RCT |
| **2** | **Allocation concealment**  Were participants/ raters unable to determine what intervention a participant would get until they were assigned? | + = yes (e.g. electronic / independent)  - = no (e.g. not sealed envelopes)  ? = unclear or not reported. NA = if not RCT |
| **3** | **Potential for baseline confounding of intervention effect**  Did treatment groups have similar characteristics? If not, could this confound effect of intervention? | + = no  - = yes; scored if clear factor influencing inflammation at baseline assessment |
| **4** | **Potential for post-baseline confounding intervention effect**  Did treatment groups have similar characteristics? If not, could this confound effect of intervention? | + = no  - = yes; scored clear factor influencing inflammation after baseline |
| **5** | **Blinding (participant & intervention)**  Were participants and those delivering intervention blinded to assigned intervention? | + = both blinded  ? = either/both were not blinded  - = either/both unblinded AND affected between-group efficacy. NA = one group |
| **6** | **Intention-to-treat analysis applied (ITT)**  Whether all participants *assigned* to intervention were analysed (or mITT; exclusion of participants with no outcomes at all) | + = ITT / mITT excluding minimal number of participants  ? = >5% patients excluded without bias correction  - = not ITT |
| **7** | **Equal treatment**  Did groups receive the same treatment, including monitoring, follow up type/length etc. | + = no evidence of different methods between groups  ? = minor differences that were accounted for in results  - = differences NA = one arm trials |
| **8** | **Appropriateness of outcomes measured**  1) groups treated differently in terms of outcomes assessed  2) multiple analyses used to assess the same outcome | + = no  ? = either/both but justification provided  - = either/both without justification |
| **9** | **Deviations from protocol / pre-specification**  Did study deviate from plans in design, treatment, or analysis? | + = no evidence of deviation that may have influenced outcomes  ? = no evidence of pre-specified methods/outcomes  - = evidence of deviation that may have influenced efficacy outcomes |
